# Veterans at High Risk for Post–COVID-19 Suicide Attempts or Other Self-Directed Violence

**DOI:** 10.1001/jamanetworkopen.2025.0061

**Published:** 2025-03-04

**Authors:** David P. Bui, Meike Niederhausen, Alex W. Hickok, Diana J. Govier, Mazhgan Rowneki, Jennifer C. Naylor, Eric Hawkins, Edward J. Boyko, Theodore J. Iwashyna, Elizabeth M. Viglianti, George N. Ioannou, Jason I. Chen, Denise M. Hynes

**Affiliations:** 1 Center to Improve Veteran Involvement in Care, VA Portland Health Care System, Portland, Oregon; 2 Portland State University School of Public Health, Oregon Health & Science University, Portland; 3 Department of Psychiatry and Behavioral Sciences, School of Medicine, Duke University, Durham, North Carolina; 4 VISN 6 Mental Illness Research, Education and Clinical Center, Durham, North Carolina; 5 Durham Veteran Administration Health Care Services, Durham, North Carolina; 6 Center of Innovation for Veteran-Centered and Value-Driven Care, VA Puget Sound HCS, Seattle, Washington; 7 Center of Excellence in Substance Addiction Treatment and Education, VA Puget Sound HCS, Seattle, Washington; 8 Department of Psychiatry and Behavioral Sciences, University of Washington, Seattle; 9 Seattle Epidemiologic Research Information Center, VA Puget Sound HCS, Seattle, Washington; 10 VA Center for Clinical Management Research, Ann Arbor VA, Ann Arbor, Michigan; 11 Department of Medicine, School of Medicine, Johns Hopkins University, Baltimore, Maryland; 12 School of Public Health, Johns Hopkins University, Baltimore, Maryland; 13 Department of Medicine, University of Michigan Medical School, Ann Arbor; 14 Division of Pulmonary and Critical Care Medicine, Department of Internal Medicine, University of Michigan, Ann Arbor; 15 Division of Gastroenterology, Department of Medicine, University of Washington, Seattle; 16 College of Health, Oregon State University, Corvallis; 17 Center for Quantitative Life Sciences, Oregon State University, Corvallis; 18 School of Nursing, Oregon Health and Science University, Portland

## Abstract

**Importance:**

US veterans have a higher risk of suicide than the general civilian population. Research suggests that COVID-19 infection is associated with increased risk of suicide attempts or other forms of self-directed violence (SDV) among veterans.

**Objective:**

To identify subgroups of veterans with high risk of post–COVID-19 suicide attempts or SDV.

**Design, Setting, and Participants:**

This is a retrospective cohort study conducted using data from the Veteran Health Administration (VHA). Participants included VHA enrollees with a first case of COVID-19 between May 1, 2021, and April 30, 2022, residing in the 50 states or Washington, DC.

**Exposure:**

COVID-19 infection.

**Main Outcomes and Measures:**

The main outcome was a suicide attempt or SDV 12 months after COVID-19 infection. Latent class analysis was used to identify subgroups. Outcome rates and 95% CIs per 10 000 veterans were calculated. Multinomial regressions were used to model outcome risk and marginal risk ratios with 99.5% CIs to compare outcome risk across latent classes.

**Results:**

The cohort included 285 235 veterans with COVID-19 and was predominantly male (248 118 veterans [87.0%]) and younger than 65 years (171 636 veterans [60.2%]). Chronic pain (152 788 veterans [53.6%]), depression (98 093 veterans [34.4%]), and posttraumatic stress disorder (79 462 veterans [27.9%]) diagnoses were common. The 12-month outcome rate was 73.8 events per 10 000 (95% CI, 70.7-77.0 events per 10 000). Two latent classes with high rates of suicide attempt or SDV were identified. The first high-risk subgroup (46 693 veterans [16.4%]) was older (34 472 veterans [73.8%] aged ≥65 years) and had a high prevalence of physical conditions (43 329 veterans [92.8%] had hypertension, and 36 824 veterans [78.9%] had chronic pain); the 12-month outcome rate was 103.7 events per 10 000 (95% CI, 94.7-113.3 events per 10 000). The second high-risk subgroup (82 309 veterans [28.9%]) was generally younger (68 822 veterans [83.6%] aged <65 years) with a lower prevalence of physical conditions but high prevalence of mental health conditions (61 367 veterans [74.6%] had depression, and 50 073 veterans [60.8%] had posttraumatic stress disorder); the 12-month outcome rate was 162.9 events per 10 000 (95% CI, 154.5-171.8 events per 10 000), and compared with the lowest risk subgroup, the 12-month risk of suicide attempts or SDV was 14 times higher in this subgroup (risk ratio, 14.23; 99.5% CI, 10.22-19.80).

**Conclusions and Relevance:**

In this cohort study of veterans with COVID-19, 2 veteran subgroups with distinct health profiles had high rates of suicide attempts and SDV, suggesting that different groups may require different approaches to suicide prevention after COVID-19.

## Key Points

**Question:**

Which subgroups of veterans are at high risk for suicide attempt or other forms of self-directed violence (SDV) after COVID-19 infection?

**Findings:**

By use of a cohort study design and latent class analysis, among a total of 285 235 veterans with COVID-19, 2 subgroups with high rates of post–COVID-19 suicide attempts and SDV were identified. The subgroups had distinct demographics, health profiles, and health care utilization.

**Meaning:**

Given the increased risk of suicide attempt and SDV after COVID-19, these results could inform ways to tailor suicide prevention to veterans at highest risk.

## Teaser

This cohort study examines characteristics of US veterans who are at high risk of suicide attempts or other self-directed violence after COVID-19 infection.

## Introduction

In 2022, 18 veterans died by suicide each day in the US, making suicide the 12th leading cause of death among US veterans.^1^ Previously, we found the risk of suicide attempts and other self-directed violence (SDV) and depressive symptoms was higher among veterans with COVID-19 than uninfected comparators.^2,3^ Other researchers have found similar links between COVID-19 and subsequent mental health sequalae in the general population, including incident depression, anxiety, and neurocognitive declines.^4,5,6^

As a population, veterans have a high prevalence of factors associated with suicide.^7^ COVID-19 is especially relevant to veteran mental health because infection can cause new conditions and exacerbate preexisting conditions associated with suicide risk among veterans.^4,6,8^ Nearly 1 in 10 veterans hospitalized for COVID-19 go on to develop new psychiatric disorders,^9^ increasing the risk of co-occurring mental health conditions and risk factors.^10,11^ Chronic pain, a highly prevalent risk factor among veterans,^8,12^ can be worsened by COVID-19 neurologic and musculoskeletal sequalae.^4,13,14^ Further neuroinflammation caused by COVID-19 may exacerbate suicide risk in veterans with traumatic brain injuries and multiple traumas.^8,15,16,17,18^ Given the high incidence of COVID-19 and high risk of suicide among veterans, understanding heterogeneity of post–COVID-19 suicide risk in this population may inform new prevention strategies.

The Veteran Health Administration (VHA) provides health care to nearly 6 million veterans annually and prioritizes suicide prevention with robust surveillance and prevention programs. VHA has implemented measures to promote secure firearm storage, expand crisis intervention services, fund clinical innovations, and tailor prevention services to veteran subpopulations.^19^ In addition to the aforementioned risk factors, suicide risk has been shown to vary by age, race, gender, sexual orientation, and rurality, and prevention strategies are most effective when matched to veterans’ risk level and needs.^20,21^ Because there is no single cause of suicide, multivariate analyses may help uncover important combinations of risk factors or patient profiles to inform more tailored and sophisticated prevention strategies.^20,21^ The goal of this study was to use electronic health records (EHRs) to identify subgroups of veterans with COVID-19 at high risk for suicide attempts and SDV to inform suicide prevention in the VHA.

## Methods

### Study Design, Setting, and Participants

We used a retrospective cohort study design and latent class analysis (LCA) to identify distinct classes (ie, subgroups) of VHA patients and assess their risk of suicide attempts and SDV after COVID-19 infection. LCA is a type of finite mixture modeling used to identify mutually exclusive, unmeasured classes in a population given a set of measured categorical variables.^22,23,24,25^ LCA models have been applied in suicide and mental health research to subgroup or risk stratify patients by anxiety and depressive symptoms, pain histories, health behaviors, comorbidities, and even toxicology measurements.^26,27,28,29,30,31^

We assembled a nationwide cohort of VHA enrollees with a first case of COVID-19 between May 1, 2021, and April 30, 2022, based on positive polymerase chain reaction tests for SARS-CoV-2 or reported to the VHA, as described elsewhere.^32^ Because circulating viral strains and access to care have changed since the beginning of the pandemic, we chose to study the second year of the pandemic to reflect more recent conditions and allow for sufficient follow-up time. To minimize missingness in EHR data, our cohort was restricted to veterans assigned to a primary care team or who had a primary care visit in the 2 years prior to infection. In addition, we excluded veterans with missing or out of range height, weight, or age data because of quality concerns, as well as veterans with missing zip codes or residing outside of Washington, DC, or the 50 states. eFigure 1 in Supplement 1 shows a flow diagram of exclusions.

Institutional review boards at VHA facilities in Seattle, Washington; Portland, Oregon; Durham, North Carolina; Ann Arbor, Michigan; and Palo Alto, California, reviewed and approved study protocols and activities. A waiver of informed consent was granted for this study because this was a retrospective study using existing data, in accordance with 45 CFR §46. We followed the Strengthening the Reporting of Observational Studies in Epidemiology (STROBE) reporting guidelines in preparing this report.

### Data Sources

Patient demographics, health conditions, and heath care utilization data were extracted from the VHA Corporate Data Warehouse, a central repository of EHR data from all VHA facilities. COVID-19–related patient data, such as vaccinations and disease severity, were obtained from the VHA COVID-19 Shared Data Resource, which was established to facilitate COVID-19 research and operations. Patient death dates were collected through Corporate Data Warehouse from multiple data sources (eg, VHA Death Ascertainment Centers for Medicare & Medicaid Services Vital Status File). Suicide attempts and SDV events were obtained from the VHA’s Office of Mental Health, which compiles data on veterans’ mental health, suicide attempts, and SDV events from clinical notes captured during routine care,^33^ which are reviewed by the Office of Mental Health Program Evaluation and Resource Centers.

### Variables

Given the rarity of outcomes in the cohort, our primary outcome was a composite indicator including first suicide attempt or SDV within 1 month (30 days), 6 months (180 days), or 1 year (365 days) after COVID-19 infection. SDV included reported preparatory behaviors, nonsuicidal SDV, and SDV with undetermined intent (eg, overdose). Because suicide attempts and SDV are distinct outcomes with different risk factors, we also analyzed suicide attempts and SDV events as separate outcomes to assess whether rates of event types varied by latent classes. We did not analyze suicide deaths as an outcome because cause of death data were unavailable; instead, all-cause mortality was analyzed separately.

We considered a broad range of indicators possibly associated with COVID-19 and our outcome to include in LCA models.^2,3,34^ In total, we selected 25 indicators, representing sociodemographic characteristics, physical and mental health diagnoses, number of prior health care visits, and history of chronic pain (see eTables 1 and 2 in Supplement 1 for full variable definitions and indicator selection methods). Because LCA models assume observed indicators are caused by latent class membership, we did not include upstream immutable demographics in fitting our LCA models but report their distribution.^22^ Race, ethnicity, and sex were self-reported by patients during administrative intake and clinical encounters. Data on race and ethnicity are included in this study because these data help highlight disparities in suicide risk and inform equitable suicide prevention programs in the VHA.

We used patient’s Area Deprivation Index (ADI) as a proxy for socioeconomic status, based on patient’s residential Census block.^35^ Urban vs rural designations were determined by patient residential zip code and were based on US Department of Agriculture’s rural-urban commuting area codes.^36^ Chronic pain was defined as having 1 or more inpatient pain diagnoses or 2 or more outpatient pain diagnosis in the year before infection.^37^ We used a comprehensive list of *International Statistical Classification of Diseases and Related Health Problems, Tenth Revision* codes to define pain diagnoses.^38^ Data for chronic pain diagnoses were limited to VHA-provided care. We included patients’ Care Assessment Needs (CAN) scores, a validated VHA risk score for 1-year hospitalization or mortality ranging from 0 to 99, with higher scores indicating higher risk.^39^ Continuous variables were categorized at medians or quantiles.

### Statistical Analysis

We used LCA to identify patient classes.^22,23^ We fitted a series of unadjusted LCA models with 1 to 10 class sizes and obtained model fit statistics for each model. We used the Bayes information criterion and considered Akaike information criterion, relative entropy, smallest class proportion, and interpretability to determine the best model fit and optimal number of latent classes; Bayes information criterion was plotted across all fitted models, and the model representing the plateau point was considered the optimal class size solution.^40^ Patients were assigned to the single latent class with highest posterior probability. To minimize misclassification, patients with a modal posterior probability of less than 50% were assigned to an unclassified class. Missingness among indicators used was rare (2.1% of participants were missing ADI, and 0.6% of participants were missing CAN score) and no imputation was conducted.

Stacked bar plots and radar plots were used to visually compare latent classes by select indicators used in LCA models and demographics not used in LCA models (eg, age, gender, and race). For radar plots, we included indicators highlighting variability and key differences across latent classes. We calculated unadjusted probabilities for all outcomes and estimated exact binomial 95% CIs. The unadjusted probabilities of suicide attempts and SDV were multiplied by 10 000, and unadjusted mortality probabilities were multiplied by 100 and reported as rates per veterans. To identify potential disparities, we calculated rates of suicide attempt and SDV stratified by patient characteristics, including race, sex, and ADI.

To compare outcome risk across latent classes, we used unadjusted multinomial logistic regressions to model the risk of suicide attempts and SDV, death, or no event by latent classes for each time frame. Another set of multinomial regressions was fitted with suicide attempt and SDV modeled as separate outcomes to assess the risk of each outcome type. We used fitted regression models to estimate pairwise marginal risk ratios (RRs) and 99.5% CIs to compare the risk of outcomes across all latent classes. To account for multiple comparisons, we used a Bonferroni correction and reported 99.5% CIs (10 pairwise comparisons; α = .005). Given the high mortality rate, we conducted a sensitivity analysis restricted to veterans who survived each time frame of interest.

Analyses were conducted in R statistical software version 4.4 (R Project for Statistical Computing). We used the poLCA package (version 1.6) to fit LCA models, the nnet package (version 7.3) to fit multinomial models, and the marginaleffects (version 0.21) package for estimating pairwise marginal RRs and 99.5% CIs.^41,42^

## Results

### Latent Classes

**Figure 1.**
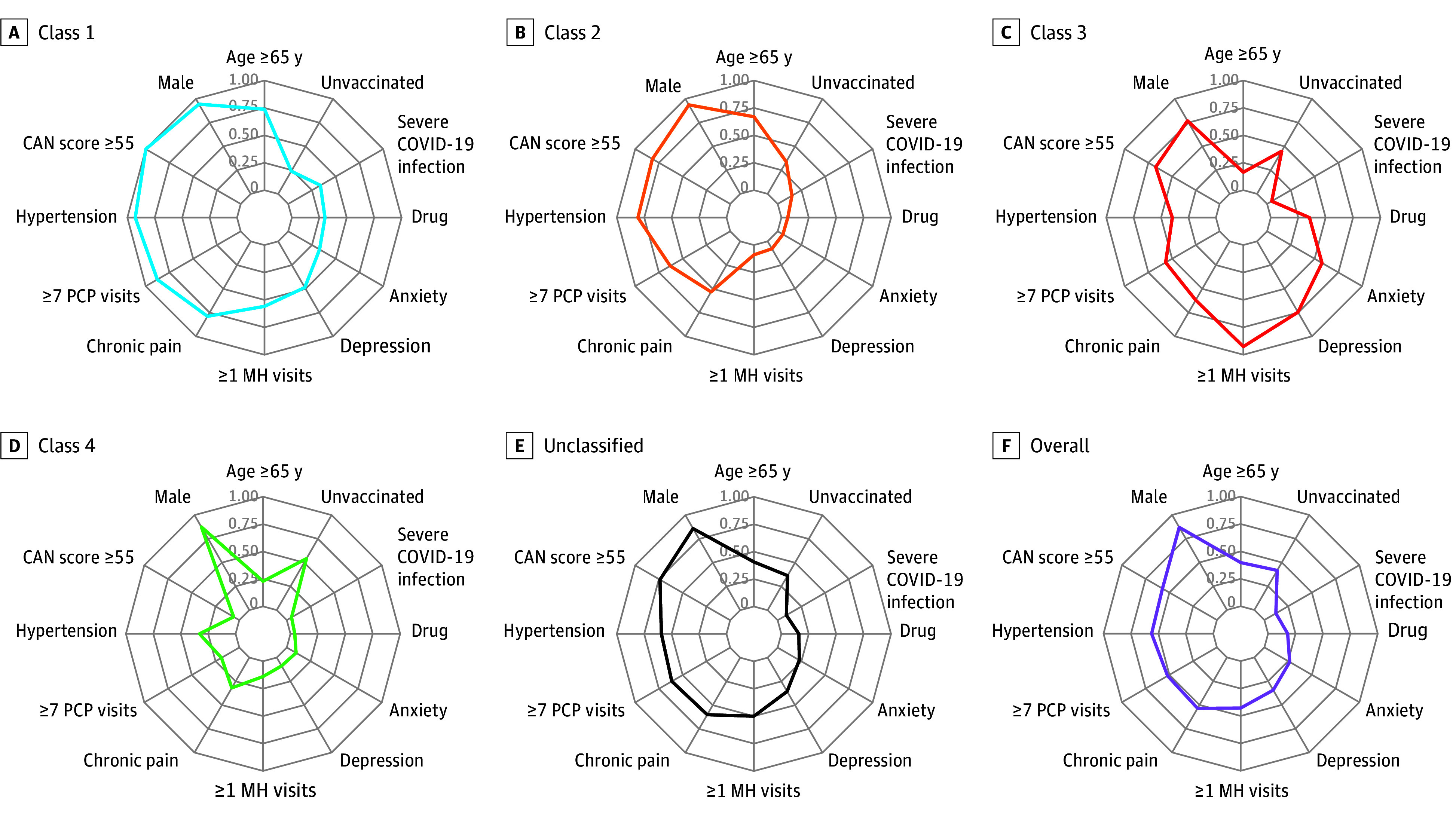
Radar Plots Summarizing Select Characteristics of Identified Latent Classes and Overall Analytic Cohort Unvaccinated refers to veterans who were not vaccinated against COVID-19. Severe COVID-19 refers to veterans who died or required hospitalization, mechanical ventilation, extracorporeal membrane oxygenation, new dialysis, vasopressors, or high-flow oxygen within 30 days. Drug refers to any nonalcohol substance use disorder diagnosis. CAN indicates care assessment needs; MH, mental health; and PCP, primary care practitioner.

Our cohort included 285 235 veterans with COVID-19 (248 118 male [87.0%]; 171 636 veterans aged <65 years [60.2%]) (Table). Chronic pain (152 788 veterans [53.6%]), depression (98 093 veterans [34.4%]), and posttraumatic stress disorder (PTSD; 79 462 veterans [27.9%]) diagnoses were common. The 4-class model resulted in the best fit (see eFigure 2 in Supplement 1). Posterior probabilities were high across all classes, with the lowest median posterior probability in class 2 (95%; see eFigure 3 in Supplement 1). There were 3666 unclassified veterans (1.3%) (Table). Radar plots shown in Figure 1 highlight characteristic differences across latent classes. See eFigures 4, 5, and 6 in Supplement 1 for stacked bar plots comparing classes on all indicators.

**Table.** Cohort Characteristics Overall and Stratified by Latent Class Assignments

Characteristic	Veterans, No. (%)					
	Overall (N = 285 235 [100%])	Class 1 (n = 46 693 [16.4%])	Class 2 (n = 66 359 [23.3%])	Class 3 (n = 82 309 [28.9%])	Class 4 (n = 86 208 [30.2%])	Unclassified (n = 3666 [1.3%])
Age group, y						
18-49	84 094 (29.5)	1550 (3.3)	3593 (5.4)	41 217 (50.1)	37 008 (42.9)	726 (19.8)
50-64	87 542 (30.7)	10 671 (22.9)	18 334 (27.6)	27 605 (33.5)	29 481 (34.2)	1451 (39.6)
65-73	58 574 (20.5)	15 480 (33.2)	20 909 (31.5)	9174 (11.1)	12 123 (14.1)	888 (24.2)
≥74	55 025 (19.3)	18 992 (40.7)	23 523 (35.4)	4313 (5.2)	7596 (8.8)	601 (16.4)
Sex						
Female	37 117 (13.0)	2587 (5.5)	4208 (6.3)	19 036 (23.1)	10 769 (12.5)	517 (14.1)
Male	248 118 (87.0)	44 106 (94.5)	62 151 (93.7)	63 273 (76.9)	75 439 (87.5)	3149 (85.9)
Race and ethnicity						
American Indian or Alaska Native	2595 (0.9)	363 (0.8)	558 (0.8)	885 (1.1)	752 (0.9)	37 (1.0)
Asian	3434 (1.2)	195 (0.4)	489 (0.7)	1202 (1.5)	1524 (1.8)	24 (0.7)
Black	57 214 (20.1)	10 262 (22.0)	11 114 (16.7)	19 762 (24.0)	15 253 (17.7)	823 (22.4)
Hispanic or Latine	19 886 (7.0)	2021 (4.3)	3224 (4.9)	7392 (9.0)	6983 (8.1)	266 (7.3)
Native Hawaiian or Pacific Islander	2807 (1.0)	385 (0.8)	591 (0.9)	901 (1.1)	900 (1.0)	30 (0.8)
White	196 853 (69.0)	32 696 (70.0)	49 270 (74.2)	52 637 (64.0)	59 782 (69.3)	2468 (67.3)
Multiracial^a^	3184 (1.1)	413 (0.9)	554 (0.8)	1179 (1.4)	1005 (1.2)	33 (0.9)
Unknown	19 148 (6.7)	2379 (5.1)	3783 (5.7)	5743 (7.0)	6992 (8.1)	251 (6.8)
Census region						
Northeast	33 900 (11.9)	5925 (12.7)	8103 (12.2)	9574 (11.6)	9842 (11.4)	456 (12.4)
Midwest	59 045 (20.7)	10 595 (22.7)	15 603 (23.5)	14 618 (17.8)	17 484 (20.3)	745 (20.3)
South	130 189 (45.6)	21 137 (45.3)	29 707 (44.8)	39 664 (48.2)	38 011 (44.1)	1670 (45.6)
West	62 101 (21.8)	9036 (19.4)	12 946 (19.5)	18 453 (22.4)	20 871 (24.2)	795 (21.7)
Urban residence	203 972 (71.5)	33 481 (71.7)	42 132 (63.5)	63 202 (76.8)	62 494 (72.5)	2663 (72.6)
Distance to nearest Veteran Health Administration medical center, miles						
≤20	135 427 (47.5)	25 049 (53.6)	27 088 (40.8)	41 557 (50.5)	39 924 (46.3)	1809 (49.3)
>20	149 808 (52.5)	21 644 (46.4)	39 271 (59.2)	40 752 (49.5)	46 284 (53.7)	1857 (50.7)
COVID-19 vaccination						
Unvaccinated	118 679 (41.6)	11 159 (23.9)	22 460 (33.8)	37 000 (45.0)	46 726 (54.2)	1334 (36.4)
Vaccinated	122 485 (42.9)	23 455 (50.2)	29 768 (44.9)	35 824 (43.5)	31 776 (36.9)	1662 (45.3)
≥1 Booster	44 071 (15.5)	12 079 (25.9)	14 131 (21.3)	9485 (11.5)	7706 (8.9)	670 (18.3)
Severe COVID-19^b^	34 482 (12.1)	15 834 (33.9)	9970 (15.0)	4015 (4.9)	4327 (5.0)	336 (9.2)
Area Deprivation Index						
1-25	42 160 (14.8)	5372 (11.5)	7580 (11.4)	13 406 (16.3)	15 306 (17.8)	496 (13.5)
26-50	82 400 (28.9)	11 400 (24.4)	17 296 (26.1)	25 412 (30.9)	27 220 (31.6)	1072 (29.2)
51-75	86 210 (30.2)	14 186 (30.4)	21 196 (31.9)	24 154 (29.3)	25 560 (29.6)	1114 (30.4)
76-100	68 449 (24.0)	14 255 (30.5)	18 941 (28.5)	17 653 (21.4)	16 683 (19.4)	917 (25.0)
Unknown	6016 (2.1)	1480 (3.2)	1346 (2.0)	1684 (2.0)	1439 (1.7)	67 (1.8)
Care Assessment Needs score^c^						
0-50 (Better health)	120 034 (42.1)	34 (0.1)	11 956 (18.0)	26 835 (32.6)	80 259 (93.1)	950 (25.9)
55-85	112 558 (39.5)	7533 (16.1)	48 877 (73.7)	48 729 (59.2)	5064 (5.9)	2355 (64.2)
90-99 (Poorer health)	50 881 (17.8)	38 800 (83.1)	5315 (8.0)	6430 (7.8)	0 (0.0)	336 (9.2)
Unknown	1762 (0.6)	326 (0.7)	211 (0.3)	315 (0.4)	885 (1.0)	25 (0.7)
Gagne score						
≤0 (Better health)	132 906 (46.6)	626 (1.3)	19 043 (28.7)	39 795 (48.3)	71 991 (83.5)	1451 (39.6)
1-3	114 226 (40.0)	14 610 (31.3)	42 232 (63.6)	41 097 (49.9)	14 217 (16.5)	2070 (56.5)
≥4 (Poorer health)	38 103 (13.4)	31 457 (67.4)	5084 (7.7)	1417 (1.7)	0 (0.0)	145 (4.0)
≥1 Mental health visit in past 12 mo	122 143 (42.8)	26 093 (55.9)	5933 (8.9)	76 237 (92.6)	12 047 (14.0)	1833 (50.0)
≥7 Primary care visits in past 24 mo	148 114 (51.9)	41 003 (87.8)	41 918 (63.2)	46 770 (56.8)	16 171 (18.8)	2252 (61.4)
≥1 Prior hospitalization in past 12 mo	43 563 (15.3)	28 725 (61.5)	7280 (11.0)	6563 (8.0)	605 (0.7)	390 (10.6)
Mental or behavioral health conditions						
Anxiety diagnosis	75 880 (26.6)	15 409 (33.0)	3656 (5.5)	47 572 (57.8)	8407 (9.8)	836 (22.8)
Depression diagnosis	98 093 (34.4)	22 671 (48.6)	5181 (7.8)	61 367 (74.6)	7570 (8.8)	1304 (35.6)
Posttraumatic stress disorder diagnosis	79 462 (27.9)	14 261 (30.5)	4111 (6.2)	50 073 (60.8)	10 049 (11.7)	968 (26.4)
Bipolar diagnosis	12 436 (4.4)	3109 (6.7)	776 (1.2)	7376 (9.0)	984 (1.1)	191 (5.2)
Schizophrenia	5748 (2.0)	2248 (4.8)	626 (0.9)	2457 (3.0)	275 (0.3)	142 (3.9)
Nonalcohol substance use disorder	50 970 (17.9)	14 090 (30.2)	3861 (5.8)	29 039 (35.3)	3394 (3.9)	586 (16.0)
Alcohol dependence	61 395 (21.5)	10 744 (23.0)	7675 (11.6)	28 794 (35.0)	13 418 (15.6)	764 (20.8)
Ever smoker	175 508 (61.5)	33 199 (71.1)	42 125 (63.5)	48 782 (59.3)	49 116 (57.0)	2286 (62.4)
Chronic pain and other comorbidities						
Chronic pain^d^	152 788 (53.6)	36 824 (78.9)	35 318 (53.2)	50 926 (61.9)	27 519 (31.9)	2201 (60.0)
Diabetes	84 760 (29.7)	29 035 (62.2)	33 702 (50.8)	13 460 (16.4)	7415 (8.6)	1148 (31.3)
Hypertension	160 243 (56.2)	43 329 (92.8)	53 615 (80.8)	32 731 (39.8)	28 393 (32.9)	2175 (59.3)
Coronary heart disease	76 995 (27.0)	36 755 (78.7)	28 257 (42.6)	7498 (9.1)	3670 (4.3)	815 (22.2)
Chronic kidney disease	63 473 (22.3)	30 978 (66.3)	20 020 (30.2)	8211 (10.0)	3606 (4.2)	658 (17.9)
Pulmonary	62 572 (21.9)	25 884 (55.4)	17 520 (26.4)	13 317 (16.2)	4993 (5.8)	858 (23.4)
Cancer	37 344 (13.1)	16 358 (35.0)	13 682 (20.6)	4816 (5.9)	2051 (2.4)	437 (11.9)
Congestive heart failure	28 739 (10.1)	21 145 (45.3)	6853 (10.3)	582 (0.7)	28 (<0.1)	131 (3.6)
Liver disease	23 155 (8.1)	8268 (17.7)	5784 (8.7)	6170 (7.5)	2632 (3.1)	301 (8.2)
Stroke or cerebrovascular	17 035 (6.0)	9074 (19.4)	5115 (7.7)	1950 (2.4)	720 (0.8)	176 (4.8)
Dementia	10 979 (3.8)	6769 (14.5)	2409 (3.6)	1381 (1.7)	273 (0.3)	147 (4.0)

^a^ Multiracial refers to more than 1 race in the electronic health record.

^b^ Refers to patients who died or required hospitalization, mechanical ventilation, extracorporeal membrane oxygenation, new dialysis, vasopressors, or high-flow oxygen within 30 days of COVID-19 infection.

^c^ Care Assessment Needs is a risk score for 1-year hospitalization or mortality ranging from 0 to 99, with higher scores indicating higher risk.

^d^ Defined as at least 1 inpatient pain diagnosis or 2 outpatient pain diagnoses in the year prior to infection.

Class 1 (older, high physical comorbidity, and high mental health burden) included 46 693 (16.4%) veterans characterized by older age (34 472 veterans [73.8%] aged ≥65 years), high physical comorbidity burden (46 333 veterans [99.2%] with CAN score ≥55; 43 329 veterans [92.8%] with hypertension), and high prevalence of chronic pain diagnoses (36 824 veterans [78.9%]). Compared with the other classes, class 1 had the highest probability of prior hospital admission (28 725 veterans [61.5%]), and severe COVID-19 (15 834 veterans [33.9%]). The prevalence of mental health diagnoses was higher among class 1 compared with the overall cohort (eg, 22 671 veterans [48.6%] with depression); 55.9% (26 093) of veterans in this class had a mental health visit in the prior 12 months (Table).

Class 2 (older, low physical comorbidity, and low mental health burden) included 66 359 veterans (23.3%) characterized by older age (44 432 veterans [67.0%] aged ≥65 years) with moderate physical comorbidities (54 192 veterans [81.7%] with CAN score ≥55) and lowest prevalence of mental health diagnoses (5181 veterans [7.8%] with major depression). Class 2 had the lowest rate of mental health use among all classes, with only 8.9% (5933 veterans) having had a mental health visit in the prior year. Despite having an age profile similar to that of class 1, class 2 was less likely to have had severe COVID-19 (9970 veterans [15.0%]) and prior hospital admissions (7280 veterans [11.0%]).

Class 3 (younger, low physical comorbidity, and high mental health burden) included 82 309 veterans (28.9%) characterized by younger age (68 822 veterans [83.6%] aged <65 years), high mental health care use (76 237 veterans [92.6%] had ≥1 mental health visit), and a high prevalence of mental health comorbidities (61 367 veterans [74.6%] had major depression diagnoses, and 50 073 veterans [60.8%] had PTSD). Chronic pain was also highly prevalent in this class (50 926 veterans [61.9%]). Class 3 had the greatest proportion of female veterans (19 036 veterans [23.1%]) and the lowest proportion of White veterans (52 637 veterans [64.0%]). The prevalence of nonalcohol drug use disorder (29 039 veterans [35.3%]) and alcohol dependence (28 794 veterans [35.0%]) was highest in class 3.

Class 4 (younger, low physical comorbidity, and low mental health burden) included 86 208 veterans (30.2%) who were younger (66 489 veterans [77.1%] aged <65 years), generally healthy (80 259 veterans [93.1%] with CAN score <50), with a low prevalence of mental health diagnoses (7570 veterans [8.8%] with major depression), and the lowest prevalence of chronic pain (27 519 veterans [31.9%]). Class 4 also had generally low health care utilization, with 0.7% (605 veterans) having had a prior inpatient admission and less than one-fifth (16 171 veterans [18.8%]) having had 7 or more primary care visits in the prior 2 years.

### Suicide Attempts and Other SDV Outcomes

**Figure 2.**
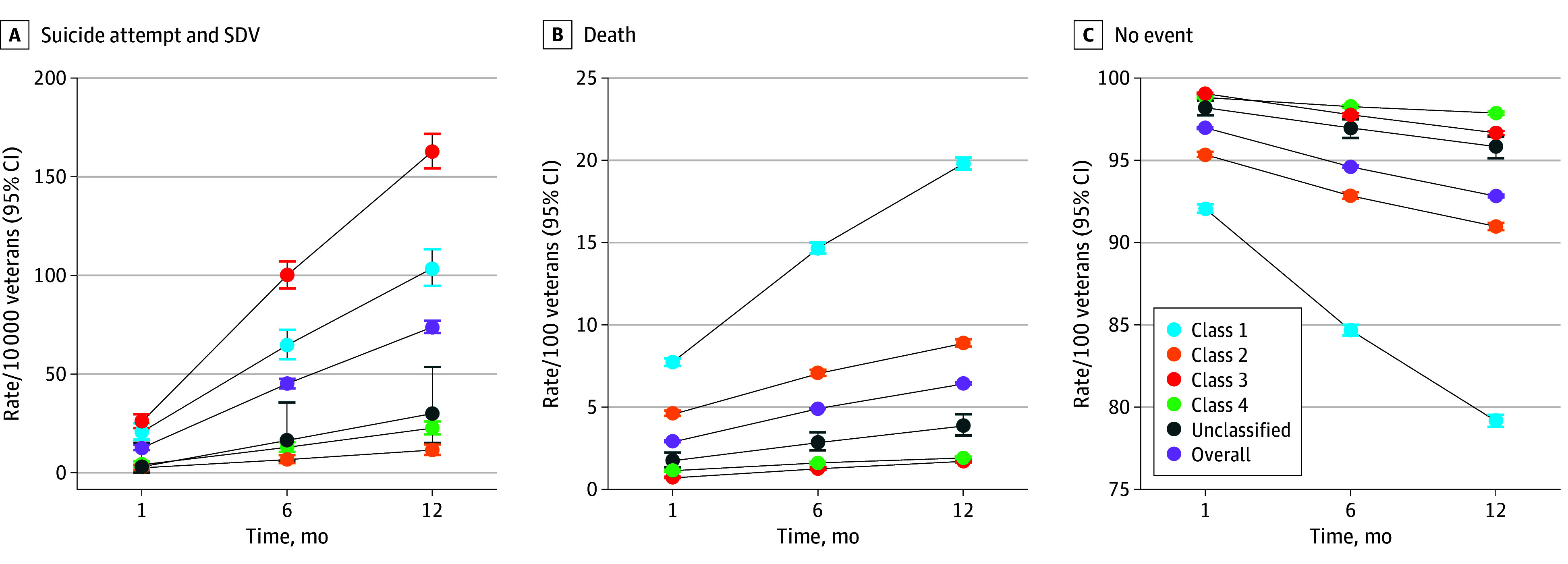
Risk of Suicide Attempt or Other Self-Directed Violence (SDV) and Death After COVID-19 Infection by Latent Classes and Overall Cohort Error bars denote 95% CIs.

During the 12 months after COVID-19 infection, 2106 veterans experienced a suicide attempt or SDV event, for a rate of 73.8 events per 10 000 veterans (95% CI, 70.7-77.0 events per 10 000) (Figure 2; eAppendix in Supplement 1). Suicide attempts accounted for 53% of events (eTable 3 in Supplement 1). Among the 2106 suicide attempts and SDV events observed within 12 months, 86.7% were among class 1 (23.0%) and class 3 (63.7%) veterans (eTable 4 in Supplement 1). Classes 2 and 4 had the lowest suicide attempt and SDV rates across all 3 time periods, with 12-month rates of 11.5 events per 10 000 (95% CI, 9.0-14.3 events per 10 000) for class 2 and 22.5 events per 10 000 (95% CI, 19.5-25.9 events per 10 000) for class 4. Classes 1 and 3 had the highest event rates over all 3 time periods, with 12-month rates of 103.7 events per 10 000 (95% CI, 94.70-113.3 events per 10 000) for class 1 and 162.9 events per 10 000 (95% CI, 154.5-171.8 events per 10 000) for class 3.

One month after infection, the relative risk of suicide attempt and SDV was comparable among veterans in classes 3 and 1 (RR, 1.26; 99.5% CI, 0.89-1.78) (eTable 5 in Supplement 1). At 6 months, the RR of suicide attempt and SDV was significantly higher for class 3 than class 1 (RR, 1.55; 99.5% CI, 1.28-1.87), and remained elevated at 12 months (RR, 1.57; 99.5% CI, 1.36-1.82). Overall, the risk of suicide attempt and SDV was highest in class 3, with a relative risk 14 times higher than that of the lowest risk (class 2) at 12 months (RR, 14.23; 99.5% CI, 10.22-19.80). Among the lower risk classes, the risk of suicide attempt and SDV for class 4 was 94% higher than class 2 at 6 months (RR, 1.94; 99.5% CI, 1.18-3.20) and remained higher at 12 months (RR, 1.96; 99.5% CI, 1.34-2.87).

**Figure 3.**
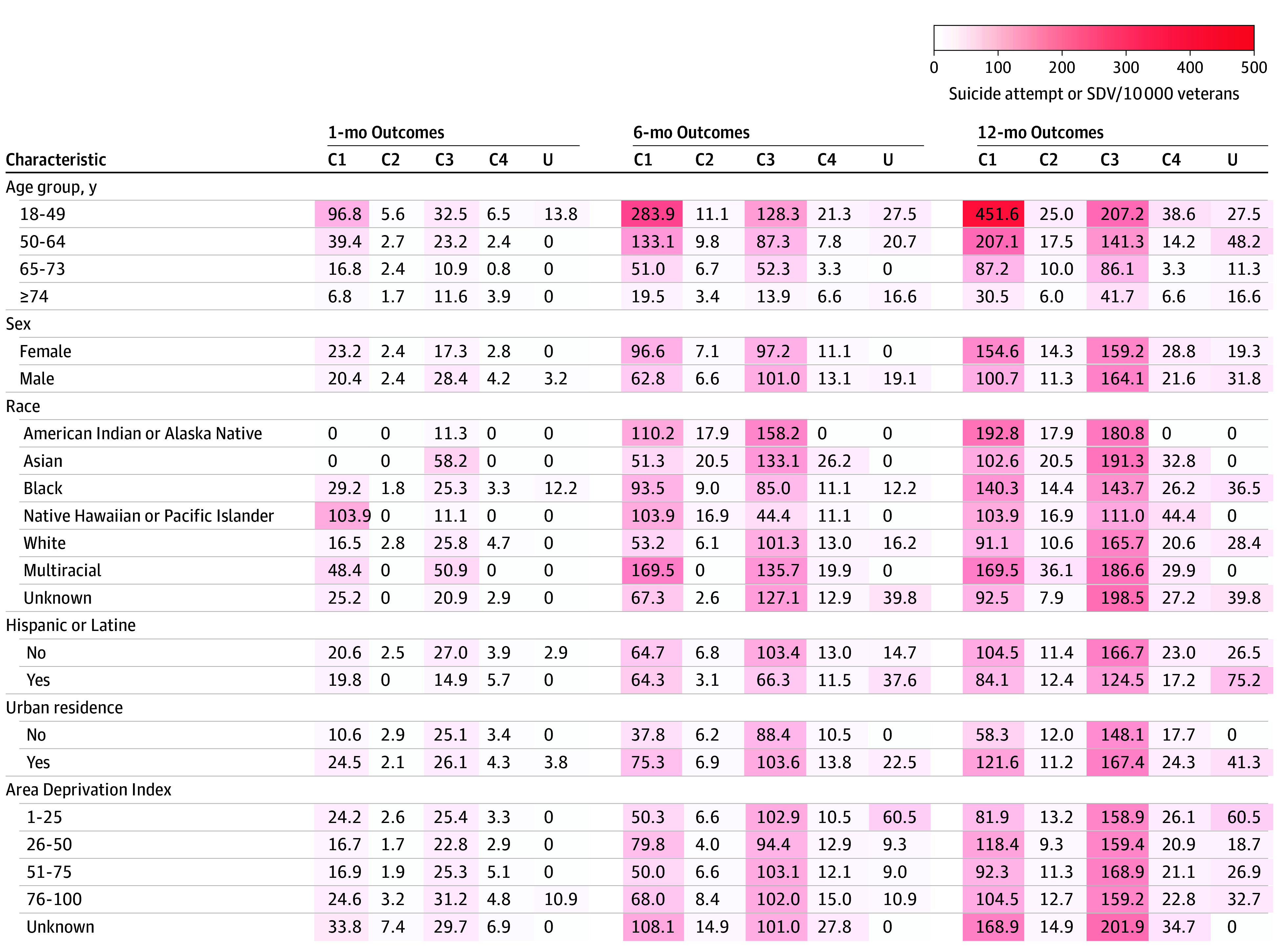
Heatmap of Risk of Suicide Attempt or Other Self-Directed Violence (SDV) Per 10 000 Veterans After COVID-19 Infection, Stratified by Sociodemographic Variables and Latent Classes C1 indicates class 1; C2, class 2; C3, class 3; C4, class 4; and U, unclassified.

In stratified analyses, we found that female veterans generally had higher risk of suicide attempt and SDV than male veterans, especially in class 1, where the 12-month rate among female veterans was 154.6 events per 10 000 (95% CI, 110.7-209.9 events per 10 000) compared with 100.7 events per 10 000 (95% CI, 91.6-110.4 events per 10 000) among male veterans (Figure 3). Outcome rates were higher in all minoritized racial groups. The highest 12-month outcome rates were observed in American Indian or Alaska Native veterans in class 1 (192.8 events per 10 000; 95% CI, 77.9-393.3 events per 10 000) and Asian veterans in class 3 (191.3 events per 10 000; 95% CI, 121.7-285.7 events per 10 000). Veterans residing in urban areas tended to have higher or similar rates compared with non–urban-residing veterans. Rates of suicide attempt and SDV increased with poorer health, particularly in class 3 where veterans with CAN scores greater than 90 had extremely high 12-month rates (541.2 events per 10 000; 95% CI, 487.2-599.3 events per 10 000) (eFigure 7 in Supplement 1).

In analyses of suicide attempts and SDV as separate outcomes, we observed similar results and associations between latent classes and outcomes (eTable 4, eTable 5, and eFigure 8 in Supplement 1). In sensitivity analyses restricted to veterans who remained alive through follow-up, we saw no changes in inferences (eTable 6 and eFigure 9 in Supplement 1).

## Discussion

In this cohort study using LCA, we identified 4 veteran classes with varying risk of post–COVID-19 suicide attempts and SDV. Two high-risk subgroups of veterans—which accounted for 87% of observed suicide attempt and SDV events—included an older subgroup characterized by high physical, mental health, and pain conditions (class 1), and a younger subgroup characterized by a high burden of mental health comorbidities and a larger proportion of female veterans (class 3). Although COVID-19 may be associated with increased overall risk of suicide attempt and SDV among veterans,^2,3^ these results suggest the risk of suicide attempts and SDV after infection may be concentrated in 2 subgroups of veterans. Stratified analyses by sex and race revealed stark disparities in suicide attempt and SDV risk among female veterans of minoritized racial groups, particularly among American Indian or Alaska Native and Asian veterans.

Chronic health conditions, pain, and age may be the important risk factors for suicide attempts and SDV in class 1 veterans.^43^ Within the context of the interpersonal theory of suicide, chronic conditions causing physical and functional impairments may contribute to increased perceived burdensomeness, leading to suicidal ideation.^44,45,46^ Class 1 veterans were frailer (high CAN scores), with high rates of prior hospitalizations and severe COVID-19. Severe illnesses and hospitalizations can be traumatizing and destabilize mental health.^47^ The high prevalence of chronic pain in this class may further contribute to increased risk for suicide in class 1.^14,48^ In addition to these comorbidities, functional impairments caused by acute COVID-19 and heightened social isolation among seniors during the pandemic could be further reasons for the high risk of suicide attempt and SDV in class 1 veterans.^49,50,51^

Class 3 had the greatest proportion of female veterans and the highest risk of suicide attempt and SDV, which is consistent with reports that female veterans have a higher rate of suicide attempts and ideation than male counterparts.^52^ Evidence suggests that female gender is associated with more-severe post–COVID-19 sequelae,^53^ which could lead to further perceived burdensomeness or capability for suicide in this class. Veterans in class 3 had high rates of mental health conditions associated with thwarted belongingness (depression), perceived burdensomeness (alcohol and nonalcohol drug dependence), and capability for suicide (PTSD).^44,54^ Although both class 1 and 3 veterans have multiple risk factors for thwarted belongingness, perceived burdensomeness, and capability for suicide, the interactions among these risk factors are likely underlying the high rates of suicide attempt and SDV events.^44^

Despite a high risk of suicide attempt and SDV, class 1 veterans had relatively low rates of prior mental health visits (55.9%) compared with class 3 veterans (92.6%), highlighting a potential gap in mental health care in class 1 veterans. Given the high rate of primary and inpatient care visits among class 1 veterans, increasing suicide screening in medical care settings could reduce suicide risk in this population.^55^ Although interventions have largely focused on routine suicide screening, implementing suicide screenings after illnesses like COVID-19 may be warranted, similar to screenings after other major health diagnoses.^56^

In stratified analyses, we found the highest rate of suicide attempt and SDV among veterans in sex and racial minoritized groups, particularly in female, American Indian or Alaska Native, and Asian veterans in classes 1 and 3. These findings are consistent with broader national trends from the most recent Annual Veteran Suicide Prevention Report,^1^ pointing to the need for special attention to how intersectional identities are associated with differential risk after life stressors such as COVID-19 infection. Female veterans face unique risk factors for suicide compared with male counterparts, including higher rates of sexual harassment, assault, and abuse, greater risk for PTSD, and lack of supportive relationships.^57,58,59^ Notably concerning is that female veterans with prior suicide attempts and military sexual trauma are less likely to seek VHA care for mental health symptoms.^60^ Given these risk factors, trauma-informed prevention for female veterans may be more effective, and educating non-VHA practitioners on the unique needs of female veterans is critical. Suicide rates among American Indian or Alaska Native veterans have grown substantially in recent decades, and the high risk in this group was reflected in our findings.^61^ Notably, although Mohatt et al^62^ found younger American Indian or Alaska Native veterans to be at highest risk for suicide, class 1 was generally older, suggesting risk factors that characterize class 1 (ie, frailty and chronic pain) may be underlying the risk of suicide among American Indian or Alaska Native veterans in this older subgroup. Given the unique historical traumas and contemporary inequities faced by American Indian or Alaska Native communities, there is a critical need to tailor and expand prevention programs for this high-risk subgroup.

Although class 3 veterans had high rates of mental health care utilization, disruptions in care during the pandemic may have resulted in unmet mental health needs, patients leaving care, or patients being lost to follow-up.^63,64^ Further research is warranted to understand the extent of disrupted care and which interventions may be needed to reengage high-risk patients with COVID-19. Given the high rates of depression and PTSD among this subgroup, suicide prevention efforts may need to include routine monitoring of suicidal ideation and/or suicide risk with specific questions or questionnaires, such as the Patient Health Questionnaire–9, to identify elevations in suicide risk and the need for timely clinical interventions.^65,66^ Given the heterogeneity in risk by race and sex in both high-risk classes, further research and evaluation on tailored and culturally appropriate interventions will be needed.^21,58,61^

### Limitations

Our study results and inferences may be subject to several limitations. During our analytic time frame, home antigen testing became more common and fewer patients were testing in VHA facilities. This limits inferences to patients who would have sought testing in VHA facilities despite the availability of home testing. Mild cases of COVID-19 are unlikely to be reported to the VHA; therefore, our cohort likely represented cases of more-severe illness. Our LCA models did not include all important indicators of suicide attempt and SDV risk, such as measures of loneliness, housing insecurity, depressive symptoms, and other psychosocial measures.^11,67,68,69,70^ We also did not include prescription and medication use in LCA models. Inclusion of these variables could improve subgroup identification and should be further explored; however, using measurements that are harder to capture may reduce usability of models for risk stratification in health care settings.^71^ Our LCA models were also fitted with only baseline data; given that patient characteristics may change over time, it is possible that class membership may also vary temporally but this was not assessed.

## Conclusions

In this cohort study of veterans with COVID-19, we identified 2 subgroups of veterans with high risk of post–COVID-19 suicide attempts and SDV. Additional suicide screening after COVID-19 diagnosis may be warranted for these veterans. Suicide is a multifaceted public health problem requiring complex and multipronged interventions. The subgroups we identified may inform new policies and highlight potential mental health care gaps that warrant attention. In particular, we found extremely high post–COVID-19 suicide attempt and SDV risk among female, American Indian or Alaska Native, and Asian veterans, highlighting the need for culturally appropriate and tailored prevention programs.

## Notes

Supplement 1. **eTable 1.** List of all indicators used in latent class analysis models **eTable 2.** Key variable definitions and data sources **eTable 3.** Total number of suicide attempts, self-directed violence outcomes, and deaths in cohort **eTable 4.** Unadjusted count and rate of events within 1, 6, and 12 months of infection stratified by latent class assignment **eTable 5.** Pairwise suicide attempt and other self-directed violence risk ratios and 99.5% confidence intervals (CI) across all identified latent class groups from marginal risk estimates derived from unadjusted multinomial regression models **eTable 6.** Sensitivity analysis: unadjusted count and rate of events within 1, 6, and 12 months of infection stratified by latent class assignment among persons who survive outcome timeframe **eFigure 1.** Flow diagram of constructing analytic cohort of Veterans **eFigure 2.** Select latent class model fit statistics **eFigure 3.** Distribution of class posterior probabilities for latent class model solution **eFigure 4.** Demographics of patients stratified by four latent class solution **eFigure 5.** Comorbidity indices and healthcare utilization patterns of patients by four latent class solution **eFigure 6.** Comorbidities of patients stratified by four latent class solution **eFigure 7.** Heatmap of crude risk of suicide attempt and other self-directed violence (SDV) per 10,000 Veterans after COVID-19 infection, stratified by sociodemographic variables and other characteristics **eFigure 8.** Unadjusted rates of all outcomes, including suicide attempt, other self-directed violence (SDV), and death by latent classes and overall **eFigure 9.** Sensitivity analysis: outcome rates by latent classes among those who survive outcome timeframes **eAppendix.** Reporting on mortality outcomes across latent classes

Supplement 2.
Data Sharing Statement
