# Supplementary material for: Veterans at High Risk for Post–COVID-19 Suicide Attempts or Other Self-Directed Violence
Source: JAMA Netw Open. 2025 Mar 4;8(3):e250061. doi: 10.1001/jamanetworkopen.2025.0061 (PMC11880954; doi:10.1001/jamanetworkopen.2025.0061)
Supplement: Supplement 1. — eTable 1. List of all indicators used in latent class analysis models eTable 2. Key variable definitions and data sources eTable 3. Total number of suicide attempts, self-directed violence outcomes, and deaths in cohort eTable 4. Unadjusted count and rate of events within 1, 6, and 12 months of infection stratified by latent class assignment eTable 5. Pairwise suicide attempt and other self-directed violence risk ratios and 99.5% confidence intervals (CI) across all identified latent class groups from marginal risk estimates derived from unadjusted multinomial regression models eTable 6. Sensitivity analysis: unadjusted count and rate of events within 1, 6, and 12 months of infection stratified by latent class assignment among persons who survive outcome timeframe eFigure 1. Flow diagram of constructing analytic cohort of Veterans eFigure 2. Select latent class model fit statistics eFigure 3. Distribution of class posterior probabilities for latent class model solution eFigure 4. Demographics of patients stratified by four latent class solution eFigure 5. Comorbidity indices and healthcare utilization patterns of patients by four latent class solution eFigure 6. Comorbidities of patients stratified by four latent class solution eFigure 7. Heatmap of crude risk of suicide attempt and other self-directed violence (SDV) per 10,000 Veterans after COVID-19 infection, stratified by sociodemographic variables and other characteristics eFigure 8. Unadjusted rates of all outcomes, including suicide attempt, other self-directed violence (SDV), and death by latent classes and overall eFigure 9. Sensitivity analysis: outcome rates by latent classes among those who survive outcome timeframes eAppendix. Reporting on mortality outcomes across latent classes [file jamanetwopen-e250061-s001.pdf]

Supplemental Online Content

Bui DP, Niederhausen M, Hickok AW, et al. Veterans at high risk for post–COVID-19 suicide attempts or other self-directed violence. *JAMA Netw Open*. 2025;8(3):e250061. doi:10.1001/jamanetworkopen.2025.0061

eTable 1. List of all indicators used in latent class analysis models

eTable 2. Key variable definitions and data sources

eTable 3. Total number of suicide attempts, self-directed violence outcomes, and deaths in cohort

eTable 4. Unadjusted count and rate of events within 1, 6, and 12 months of infection stratified by latent class assignment

eTable 5. Pairwise suicide attempt and other self-directed violence risk ratios and 99.5% confidence intervals (CI) across all identified latent class groups from marginal risk estimates derived from unadjusted multinomial regression models

eTable 6. Sensitivity analysis: unadjusted count and rate of events within 1, 6, and 12 months of infection stratified by latent class assignment among persons who survive outcome timeframe

eFigure 1. Flow diagram of constructing analytic cohort of Veterans

eFigure 2. Select latent class model fit statistics

eFigure 3. Distribution of class posterior probabilities for latent class model solution

eFigure 4. Demographics of patients stratified by four latent class solution

eFigure 5. Comorbidity indices and healthcare utilization patterns of patients by four latent class solution

eFigure 6. Comorbidities of patients stratified by four latent class solution

eFigure 7. Heatmap of crude risk of suicide attempt and other self-directed violence (SDV) per 10,000 Veterans after COVID-19 infection, stratified by sociodemographic variables and other characteristics

eFigure 8. Unadjusted rates of all outcomes, including suicide attempt, other self-directed violence (SDV), and death by latent classes and overall

eFigure 9. Sensitivty analysis: outcome rates by latent classes among those who survive outcome timeframes

eAppendix. Reporting on mortality outcomes across latent classes

This supplemental material has been provided by the authors to give readers additional information about their work.

© 2025 Bui DP et al. *JAMA Network Open*.

eTable 1. List of all indicators used in latent class analysis models.

Variable Category	Variable Description	Levels
Access to Care	Distance to nearest VA medical center	≤20 miles; >20 miles
Mental Health	History of Depression diagnoses	No, Yes
Mental Health	History of Anxiety diagnoses	No, Yes
Mental Health	History of Non-alcohol Drug Dependence	No, Yes
Mental Health	History of Alcohol Dependence	No, Yes
Mental Health	PTSD Diagnoses	No, Yes
Comorbidity Indices	Care Assessment Needs Score (CAN)	<50; 55-85; 90-99
Comorbidity Indices	Gagne Index	≤0, 1-3, >3
COVID-19	Severity of COVID based on 30d outcomes	Mild; Moderate-to-severe COVID-19 including death
COVID-19	COVID-19 Vaccination status	No vaccinations; Primary series or other; ≥1 boosters
Demographics	Area Deprivation Index (ADI)	1-24 (least deprivation); 25-49; 50-74; 75-100 (most deprivation)
Demographics	Census Region	Northeast; Midwest; South; West
Demographics	Urban Resident	Not urban resident; Urban resident
Oher Conditions	History of ever smoking (tobacco)	Never smoker or missing; Ever smoker
Other Conditions	History of Pulmonary	No, Yes
Other Conditions	History of Congestive Heart Failure	No, Yes
Other Conditions	History of Chronic Kidney Disease	No, Yes
Other Conditions	History of Cancer	No, Yes
Other Conditions	History of Coronary Heart Disease	No, Yes
Other Conditions	History of Hypertension	No, Yes
Other Conditions	History of Diabetes	No, Yes
Other Conditions	Chronic Pain Diagnosis	No, Yes
Utilization	Prior PCP Visits (24 months)	<7 visits; 7+ visits
Utilization	Prior Mental Health Visits (12 months)	None, At least 1
Utilization	Prior Inpatient Admission (12 months)	None, At least 1

Indicator selection methods: We reviewed a broad range of indicators possibly associated with COVID-19 and suicide attempts and other self-directed violence to include in LCA models. We first considered all indicators for conditions and characteristics that were not rare (>10% prevalence). Among the subset of prevalent indicators, we calculated pairwise correlation coefficients to identify strongly correlated indicators (≥0.70 Spearman correlation coefficient) to exclude. Only two considered indicators-"Chronic pain" and "Outpatient pain diagnoses"- were strongly correlated; we selected the former since it included the latter. The second highest correlation was between Gagne and CAN scores (0.62) and was below our correlation threshold for exclusion, so both were included.

© 2025 Bui DP et al. *JAMA Network Open*.

eTable 2. Key variable definitions and data sources

Variables and Indicators	Definition/Values	Data Sources/Notes
Area Deprivation Index (ADI)	National percentile rank of Census block group level socioeconomic disadvantage. The index includes factors related to household income, employment, and housing. ADI is based on patient's Census block of residence at time of infection.	Kind AJH, Buckingham WR. Making Neighborhood-Disadvantage Metrics Accessible - The Neighborhood Atlas. N Engl J Med. 2018;378(26):2456-2458. doi:10.1056/NEJMp1802313.<br><br>The Geospatial Service Support Center (GSSC) and CDW Patient tables.
Care Assessment Needs (CAN) Score (1-year mortality)	An automatically generated and validated risk score for 1-year mortality developed for Veteran Health Administration (VHA) patients based on patient's electronic health record history of comorbidities, utilization, and prescriptions. Score ranges from 0-99 with higher scores indicating higher risk.	Wang L, Porter B, Maynard C, et al. Predicting risk of hospitalization or death among patients receiving primary care in the Veterans Health Administration. Med Care. 2013;51(4):368-373. doi:10.1097/MLR.0b013e31827da95a<br><br>VA Corporate Data Warehouse (CDW)
Gagne index	A validated comorbidity score for predicting 1-year mortality. Score is a combination of Charlson and Elixhauser measures. The index is automatically calculated for VHA patients based on ICD-10 diagnoses in the electronic health record.	Gagne JJ, Glynn RJ, Avorn J, Levin R, Schneeweiss S. A combined comorbidity score predicted mortality in elderly patients better than existing scores. J Clin Epidemiol. 2011;64(7):749-759. doi:10.1016/j.jclinepi.2010.10.004<br><br>VA Corporate Datawarehouse (CDW)
Severe COVID-19	Based on clinical outcomes within 30 days of COVID-19 infection. Patients who requiring mechanical ventilation, ECMO, dialysis, vasopressors, or high flow oxygen within 30 days of COVID-19 test positive date were considered severe.	COVID-19 Shared Data Resource (CSDR)
Urban/rural	Based on patient residential ZIP code and classifications based on USDA's urban-rural commuting area codes.	The Geospatial Service Support Center (GSSC) and Corporate Data Warehouse (CDW) Patient tables. Rural-urban commuting area codes: 1.0,1.1,2.0,2.1,3.0,4.1,5.1,7.1,8.1,10.1

© 2025 Bui DP et al. *JAMA Network Open*.

Census Region	Based on patient's state residence mapped to U.S. Census Region definitions.	https://www2.census.gov/programs-surveys/sahie/reference-maps/2020/us_regdiv.pdf<br><br>The Geospatial Service Support Center (GSSC) and CDW Patient tables.
**Variables and Indicators**	**Definition/Values**	**Data Sources/Notes**
Distance to nearest Veteran Affairs Medical Center (VAMC)	Straight line distance (in miles) between patient's residential Zip code centroid and nearest VA medical center (station).	The Geospatial Service Support Center (GSSC)
Chronic Pain Diagnoses	Patient flagged for chronic pain diagnoses if they had 2+ outpatient pain diagnoses or 1+ inpatient pain diagnoses in the year prior to COVID-19 infection. Pain diagnoses based on ICD-10 codes from Mayhew et al. and include diagnoses for limb/extremity pain, back pain, abdominal pain, neuropathy, and others.	Mayhew M, DeBar LL, Deyo RA, et al. Development and Assessment of a Crosswalk Between ICD-9-CM and ICD-10-CM to Identify Patients with Common Pain Conditions. J Pain. 2019;20(12):1429-1445. doi:10.1016/j.jpain.2019.05.006<br><br>Diagnoses codes from VA CDW outpatient and inpatient encounter diagnoses tables.
Comorbidities (e.g., anxiety, depression, diabetes)	Patient comorbidities based on presence of ICD-10 codes in electronic health record.	COVID-19 Shared Data Resource (CSDR)
Alcohol dependence	Based on a combination AUDIT-C scores, CPT codes, and ICD-10 diagnoses.	COVID-19 Shared Data Resource (CSDR)

© 2025 Bui DP et al. *JAMA Network Open*.

eTable 3. Total number of suicide attempts, self-directed violence outcomes, and deaths in cohort.

Outcome Type	Frequency
**Suicide Attempt or Self-directed Violence**	2,106
Suicide Attempt	1,125
Self-directed Violence*	981
**Deaths (all-cause)**	18,332

* includes preparatory behaviors, non-suicidal self-directed violence, and other self-directed violence with undetermined intent

© 2025 Bui DP et al. *JAMA Network Open*.

eTable 4. Unadjusted count and rate of events within 1, 6, and 12 months of infection stratified by latent class assignment; 95% exact binomial confidence intervals are reported.

Rates are per 10,000 for suicide-related outcomes and per 100 Veterans for mortality.

Suicide Attempt or Other Self-directed Violence (SDV)						
	1-Month Outcomes		6-Month Outcomes		12-Month Outcomes	
Class	n	Rate (95%CI)	n	Rate (95%CI)	n	Rate (95%CI)
C1	96	20.6 (16.7, 25.1)	302	64.7 (57.6, 72.4)	484	103.7 (94.7, 113.3)
C2	16	2.4 (1.4, 3.9)	44	6.6 (4.8, 8.9)	76	11.5 (9.0, 14.3)
C3	213	25.9 (22.5, 29.6)	824	100.1 (93.4, 107.1)	1341	162.9 (154.4, 171.8)
C4	35	4.1 (2.8, 5.6)	111	12.9 (10.6, 15.5)	194	22.5 (19.5, 25.9)
U	1	2.7 (0.1, 15.2)	6	16.4 (6.0, 35.6)	11	30.0 (15.0, 53.6)
Overall	361	12.7 (11.4, 14.0)	1287	45.1 (42.7, 47.6)	2106	73.8 (70.7, 77.0)
**Suicide Attempt**						
	1-Month Outcomes		6-Month Outcomes		12-Month Outcomes	
Class	n	Rate (95%CI)	n	Rate (95%CI)	n	Rate (95%CI)
C1	52	11.1 (8.3, 14.6)	164	35.1 (30.0, 40.9)	268	57.4 (50.7, 64.7)
C2	7	1.1 (0.4, 2.2)	24	3.6 (2.3, 5.4)	40	6.0 (4.3, 8.2)
C3	104	12.6 (10.3, 15.3)	441	53.6 (48.7, 58.8)	712	86.5 (80.3, 93.1)
C4	18	2.1 (1.2, 3.3)	56	6.5 (4.9, 8.4)	100	11.6 (9.4, 14.1)
U	0	-	2	5.5 (0.7, 19.7)	5	13.6 (4.4, 31.8)
Overall	181	6.3 (5.5, 7.3)	687	24.1 (22.3, 26.0)	1125	39.4 (37.2, 41.8)
**Other Self-directed Violence**						
	1-Month Outcomes		6-Month Outcomes		12-Month Outcomes	
Class	n	Rate (95%CI)	n	Rate (95%CI)	n	Rate (95%CI)
C1	44	9.4 (6.8, 12.6)	138	29.6 (24.8, 34.9)	216	46.3 (40.3, 52.8)
C2	9	1.4 (0.6, 2.6)	20	3.0 (1.8, 4.7)	36	5.4 (3.8, 7.5)
C3	109	13.2 (10.9, 16.0)	383	46.5 (42.0, 51.4)	629	76.4 (70.6, 82.6)
C4	17	2.0 (1.1, 3.2)	55	6.4 (4.8, 8.3)	94	10.9 (8.8, 13.3)
U	1	2.7 (0.1, 15.2)	4	10.9 (3.0, 27.9)	6	16.4 (6.0, 35.6)
Overall	180	6.3 (5.4, 7.3)	600	21.0 (19.4, 22.8)	981	34.4 (32.3, 36.6)
**All-cause Mortality***						
	1-Month Outcomes		6-Month Outcomes		12-Month Outcomes	
Class	n	Rate (95%CI)	n	Rate (95%CI)	n	Rate (95%CI)
C1	3604	7.7 (7.5, 8.0)	6852	14.7 (14.4, 15.0)	9244	19.8 (19.4, 20.2)
C2	3064	4.6 (4.5, 4.8)	4695	7.1 (6.9, 7.3)	5905	8.9 (8.7, 9.1)
C3	575	0.7 (0.6, 0.8)	1019	1.2 (1.2, 1.3)	1401	1.7 (1.6, 1.8)
C4	977	1.1 (1.1, 1.2)	1373	1.6 (1.5, 1.7)	1640	1.9 (1.8, 2.0)
U	64	1.7 (1.3, 2.2)	105	2.9 (2.3, 3.5)	142	3.9 (3.3, 4.5)
Overall	8284	2.9 (2.8, 3.0)	14044	4.9 (4.8, 5.0)	18332	6.4 (6.3, 6.5)

* mortality rates are per 100 Veterans; all other outcome rates are per 10,000 Veterans.

© 2025 Bui DP et al. *JAMA Network Open*.

eTable 5 Pairwise suicide attempt and other self-directed violence risk ratios and 99.5% confidence intervals (CI) across all identified latent class groups from marginal risk estimates derived from unadjusted multinomial regression models.

	1-Month		6-Month		12-Month	
Contrast	Risk Ratio	99.5% CI	Risk Ratio	99.5% CI	Risk Ratio	99.5% CI
**Suicide Attempt and Other Self-directed Violence (composite outcome)**						
C2 / C1	0.12	(0.05, 0.25)	0.10	(0.07, 0.16)	0.11	(0.08, 0.16)
C3 / C1	1.26	(0.89, 1.78)	1.55	(1.28, 1.87)	1.57	(1.36, 1.82)
C3 / C2	10.74	(5.19, 22.23)	15.10	(9.78, 23.31)	14.23	(10.22, 19.8)
C4 / C1	0.20	(0.11, 0.34)	0.20	(0.15, 0.27)	0.22	(0.17, 0.28)
C4 / C2	1.68	(0.72, 3.93)	1.94	(1.18, 3.2)	1.96	(1.34, 2.87)
C4 / C3	0.16	(0.09, 0.26)	0.13	(0.1, 0.17)	0.14	(0.11, 0.17)
U / C1	0.13	(0.01, 2.23)	0.25	(0.08, 0.8)	0.29	(0.12, 0.68)
U / C2	1.13	(0.06, 20.43)	2.47	(0.73, 8.36)	2.62	(1.06, 6.47)
U / C3	0.11	(0.01, 1.76)	0.16	(0.05, 0.52)	0.18	(0.08, 0.43)
U / C4	0.67	(0.04, 11.57)	1.27	(0.39, 4.12)	1.33	(0.56, 3.18)
**Suicide Attempt**						
C2 / C1	0.09	(0.03, 0.29)	0.10	(0.05, 0.19)	0.11	(0.07, 0.17)
C3 / C1	1.13	(0.7, 1.83)	1.58	(1.22, 2.04)	1.51	(1.23, 1.84)
C3 / C2	11.98	(4, 35.83)	15.52	(8.53, 28.26)	14.16	(9, 22.28)
C4 / C1	0.19	(0.09, 0.4)	0.19	(0.12, 0.3)	0.20	(0.15, 0.28)
C4 / C2	1.98	(0.57, 6.91)	1.89	(0.94, 3.78)	1.90	(1.13, 3.21)
C4 / C3	0.17	(0.08, 0.34)	0.12	(0.08, 0.18)	0.13	(0.1, 0.18)
U / C1	*	*	0.18	(0.03, 1.18)	0.24	(0.07, 0.84)
U / C2	*	*	1.72	(0.24, 12.49)	2.22	(0.58, 8.42)
U / C3	*	*	0.11	(0.02, 0.74)	0.16	(0.04, 0.55)
U / C4	*	*	0.91	(0.13, 6.28)	1.17	(0.32, 4.24)
**Other Self-directed Violence**						
C2 / C1	0.14	(0.05, 0.4)	0.10	(0.05, 0.2)	0.12	(0.07, 0.19)
C3 / C1	1.41	(0.85, 2.32)	1.60	(1.21, 2.12)	1.65	(1.32, 2.06)
C3 / C2	9.77	(3.69, 25.85)	15.89	(8.27, 30.53)	14.07	(8.7, 22.75)
C4 / C1	0.21	(0.09, 0.47)	0.22	(0.14, 0.35)	0.24	(0.17, 0.33)
C4 / C2	1.45	(0.46, 4.62)	2.19	(1.05, 4.6)	2.01	(1.16, 3.48)
C4 / C3	0.15	(0.07, 0.31)	0.14	(0.09, 0.21)	0.14	(0.1, 0.19)
U / C1	0.29	(0.02, 4.94)	0.49	(0.14, 1.71)	0.34	(0.11, 1.12)
U / C2	2.01	(0.1, 38.75)	4.83	(1.21, 19.33)	2.93	(0.84, 10.24)
U / C3	0.21	(0.01, 3.45)	0.30	(0.09, 1.05)	0.21	(0.06, 0.67)
U / C4	1.38	(0.08, 24.84)	2.20	(0.61, 7.99)	1.46	(0.44, 4.83)
**All-cause Mortality**						
C2 / C1	0.60	(0.56, 0.64)	0.48	(0.46, 0.51)	0.45	(0.43, 0.47)
C3 / C1	0.09	(0.08, 0.1)	0.08	(0.08, 0.09)	0.09	(0.08, 0.09)
C3 / C2	0.15	(0.13, 0.17)	0.18	(0.16, 0.19)	0.19	(0.18, 0.21)
C4 / C1	0.15	(0.13, 0.16)	0.11	(0.1, 0.12)	0.10	(0.09, 0.1)
C4 / C2	0.25	(0.22, 0.27)	0.22	(0.2, 0.24)	0.21	(0.2, 0.23)
C4 / C3	1.62	(1.4, 1.88)	1.27	(1.13, 1.43)	1.12	(1.01, 1.24)
U / C1	0.23	(0.16, 0.32)	0.20	(0.15, 0.26)	0.20	(0.16, 0.25)
U / C2	0.38	(0.27, 0.54)	0.41	(0.31, 0.53)	0.44	(0.34, 0.55)
U / C3	2.50	(1.73, 3.61)	2.31	(1.74, 3.07)	2.28	(1.79, 2.9)
U / C4	1.54	(1.08, 2.21)	1.82	(1.37, 2.4)	2.04	(1.6, 2.59)

* no suicide attempt events observed in unassigned class in 1-month timeframe

© 2025 Bui DP et al. *JAMA Network Open*.

eTable 6. Sensitivity analysis: unadjusted count and rate of events within 1, 6, and 12 months of infection stratified by latent class assignment among persons who survive outcome timeframe.

Rates are per 10,000 for suicide-related outcomes.

Class	1-Month Outcomes		6-Month Outcomes		12-Month Outcomes	
	n	Rate (95%CI)	n	Rate (95%CI)	n	Rate (95%CI)
**Suicide Attempt OR Self-directed Violence (composite outcome)**						
C1	97	22.5 (18.4,27.6)	303	76.1 (67.9,85.2)	485	129.5 (118.4,141.6)
C2	16	2.5 (1.5,4.2)	44	7.1 (5.2,9.7)	76	12.6 (10,15.8)
C3	214	26.2 (22.8,30)	825	101.5 (94.8,108.7)	1342	165.9 (157.2,175)
C4	36	4.2 (3,5.9)	112	13.2 (10.9,15.9)	195	23.1 (20,26.6)
U	1	2.8 (0.1,18)	6	16.8 (6.9,38.6)	11	31.2 (16.4,57.6)
Overall	364	13.1 (11.8,14.6)	1290	47.6 (45,50.2)	2109	79 (75.7,82.5)
**Other Self-directed Violence**						
C1	44	10.2 (7.4,13.7)	138	34.6 (29.1,40.9)	216	57.7 (50.3,65.9)
C2	9	1.4 (0.7,2.7)	20	3.2 (2,5)	36	6 (4.2,8.2)
C3	109	13.3 (11,16.1)	383	47.1 (42.5,52.1)	629	77.7 (71.8,84)
C4	17	2 (1.2,3.2)	55	6.5 (4.9,8.4)	94	11.1 (9,13.6)
U	1	2.8 (0.1,15.5)	4	11.2 (3.1,28.7)	6	17 (6.3,37)
Overall	180	6.5 (5.6,7.5)	600	22.1 (20.4,24)	981	36.8 (34.5,39.1)
**Suicide Attempt**						
C1	52	12.1 (9,15.8)	164	41.2 (35.1,48)	268	71.6 (63.3,80.6)
C2	7	1.1 (0.4,2.3)	24	3.9 (2.5,5.8)	40	6.6 (4.7,9)
C3	104	12.7 (10.4,15.4)	441	54.3 (49.3,59.5)	712	88 (81.7,94.7)
C4	18	2.1 (1.3,3.3)	56	6.6 (5,8.6)	100	11.8 (9.6,14.4)
U	0	-	2	5.6 (0.7,20.3)	5	14.2 (4.6,33.1)
Overall	181	6.5 (5.6,7.6)	687	25.3 (23.5,27.3)	1125	42.2 (39.7,44.7)

© 2025 Bui DP et al. *JAMA Network Open*.

eFigure 1. Flow diagram of constructing analytic cohort of Veterans

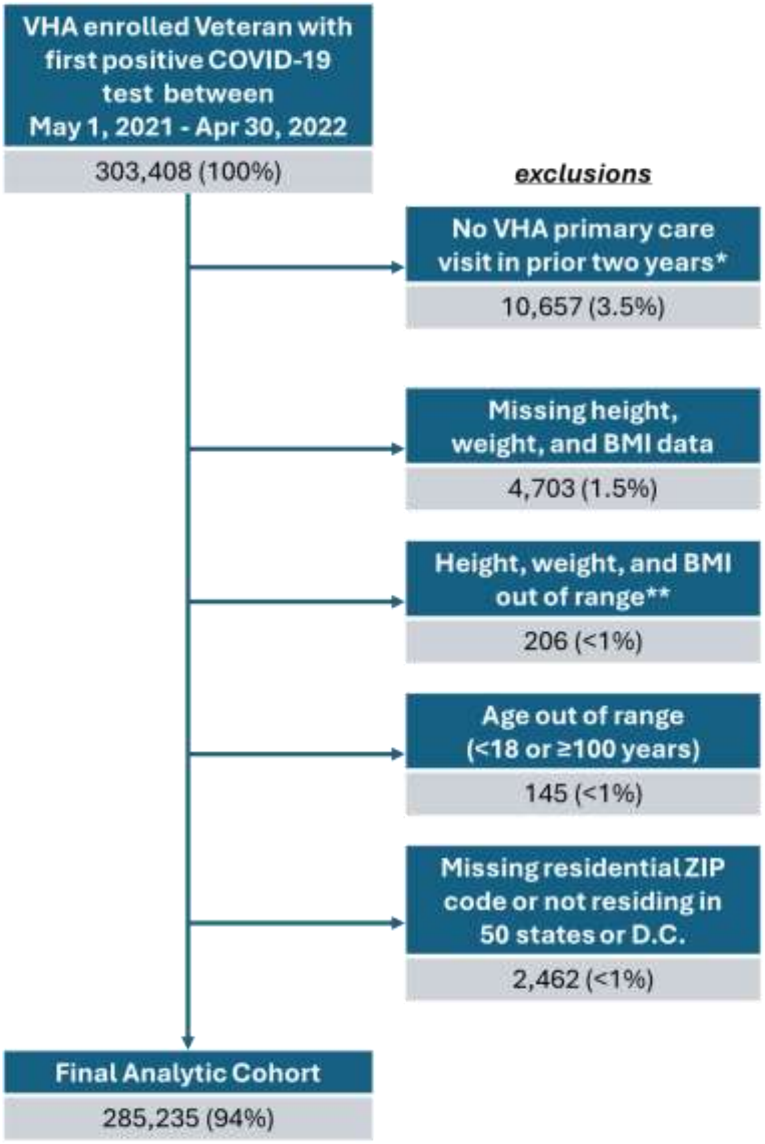

* Veterans not assigned to a primary care team were also excluded
** height exclusions: <48 inches or >84 inches; weight exclusions: <50 pounds or >700 pounds; BMI exclusions: <14 kg/m^2 or >90 kg/m^2

© 2025 Bui DP et al. *JAMA Network Open*.

eFigure 2. Select latent class model fit statistics.

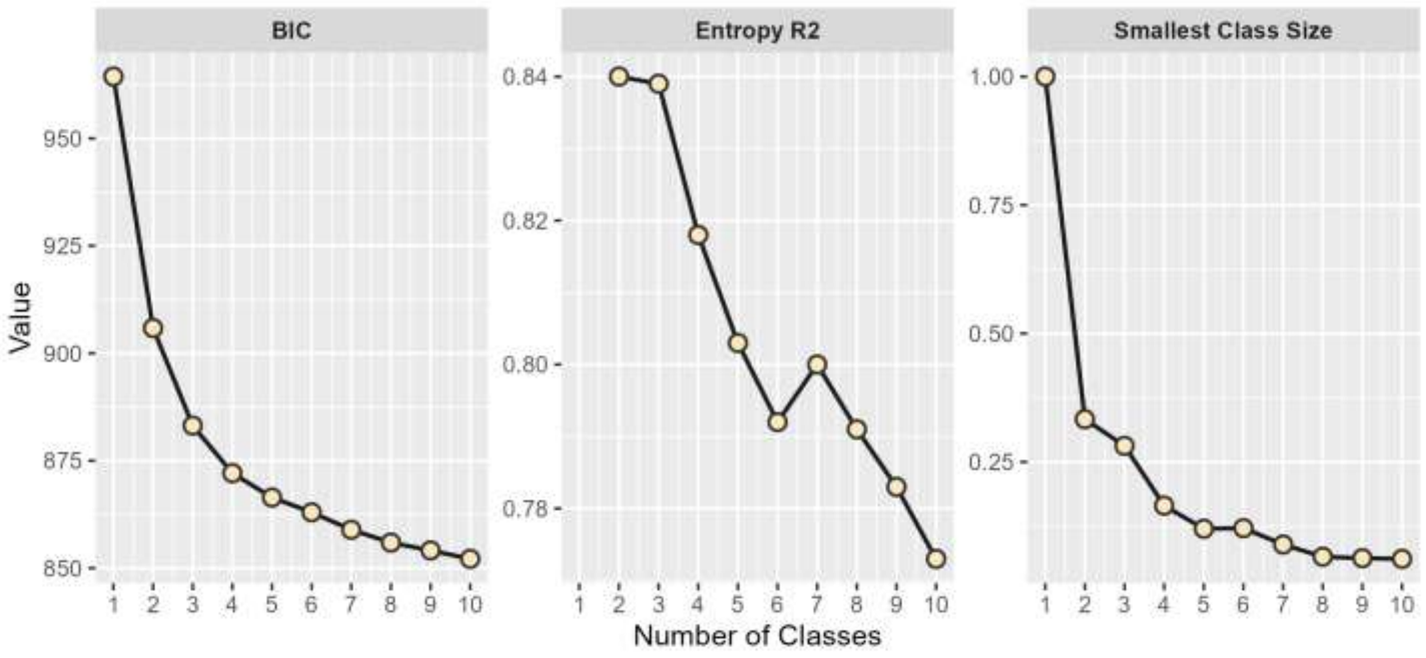

No. Class Solutions	Log Likelihood	BIC	AIC	Entropy R2	Smallest Class Proportion
1	-4821688.391	9643778.74	9643440.78	NA	100%
2	-4528836.743	9058489.96	9057803.49	0.84	33.3%
3	-4415219.397	8831669.78	8830634.79	0.84	28.1%
4	-4359684.354	8721014.21	8719630.71	0.82	16.5%
5	-4331144.319	8664348.65	8662616.64	0.80	12.0%
6	-4313694.396	8629863.32	8627782.79	0.79	12.1%
7	-4293200.87	8589290.79	8586861.74	0.80	8.9%
8	-4278034.857	8559373.27	8556595.71	0.79	6.5%
9	-4268721.429	8541160.94	8538034.86	0.78	6.3%
10	-4258681.336	8521495.26	8518020.67	0.77	6.1%

© 2025 Bui DP et al. *JAMA Network Open*.

eFigure 3. Distribution of class posterior probabilities for latent class model solution.

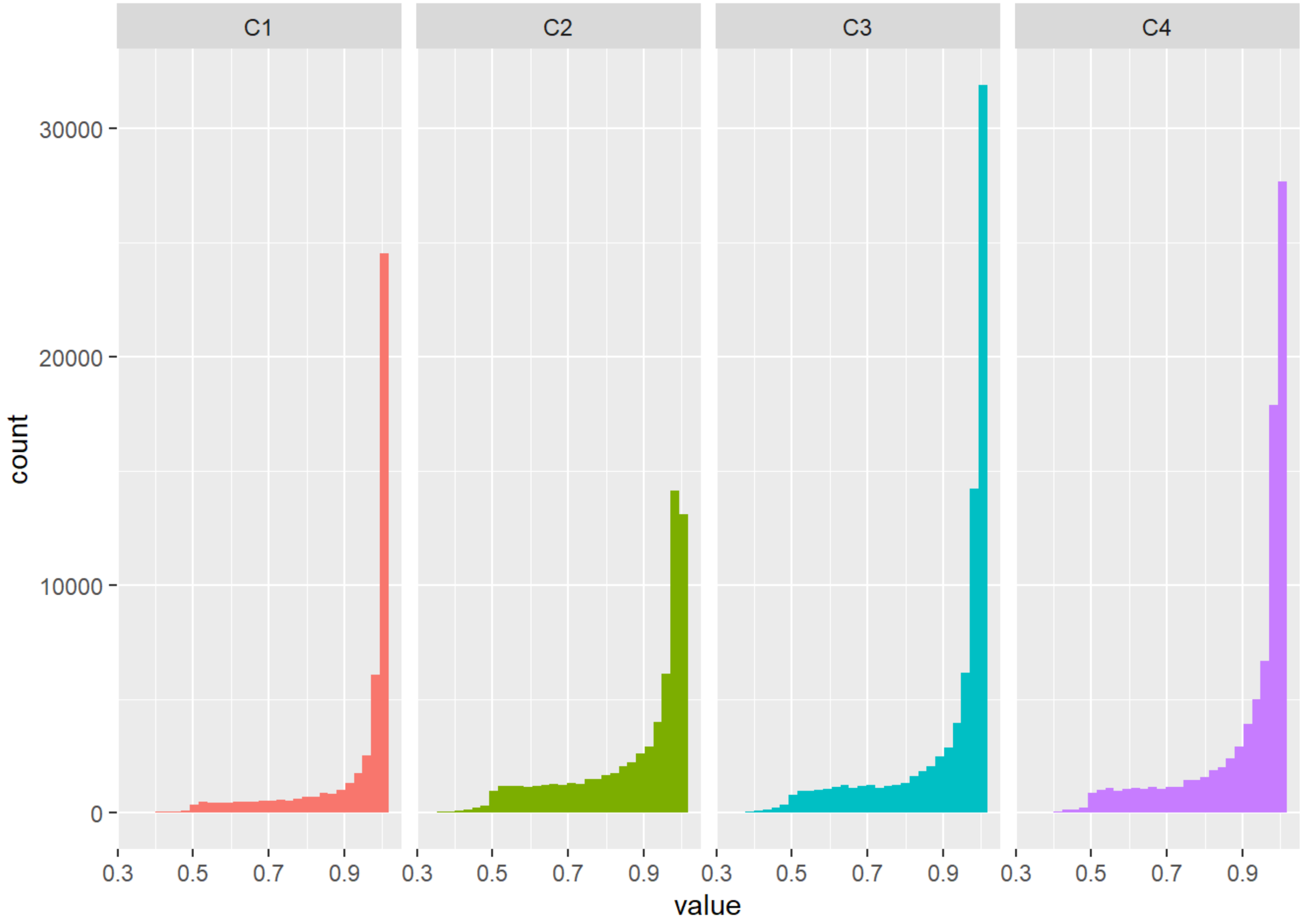

Class	Mean	Median	Min	Max	25%	75%
C1	0.926	0.995	0.339	1.000	0.918	1.000
C2	0.871	0.946	0.344	1.000	0.786	0.991
C3	0.905	0.981	0.341	1.000	0.868	0.998
C4	0.905	0.976	0.337	1.000	0.868	0.997

© 2025 Bui DP et al. *JAMA Network Open*.

eFigure 4. Demographics of patients stratified by four latent class solution.

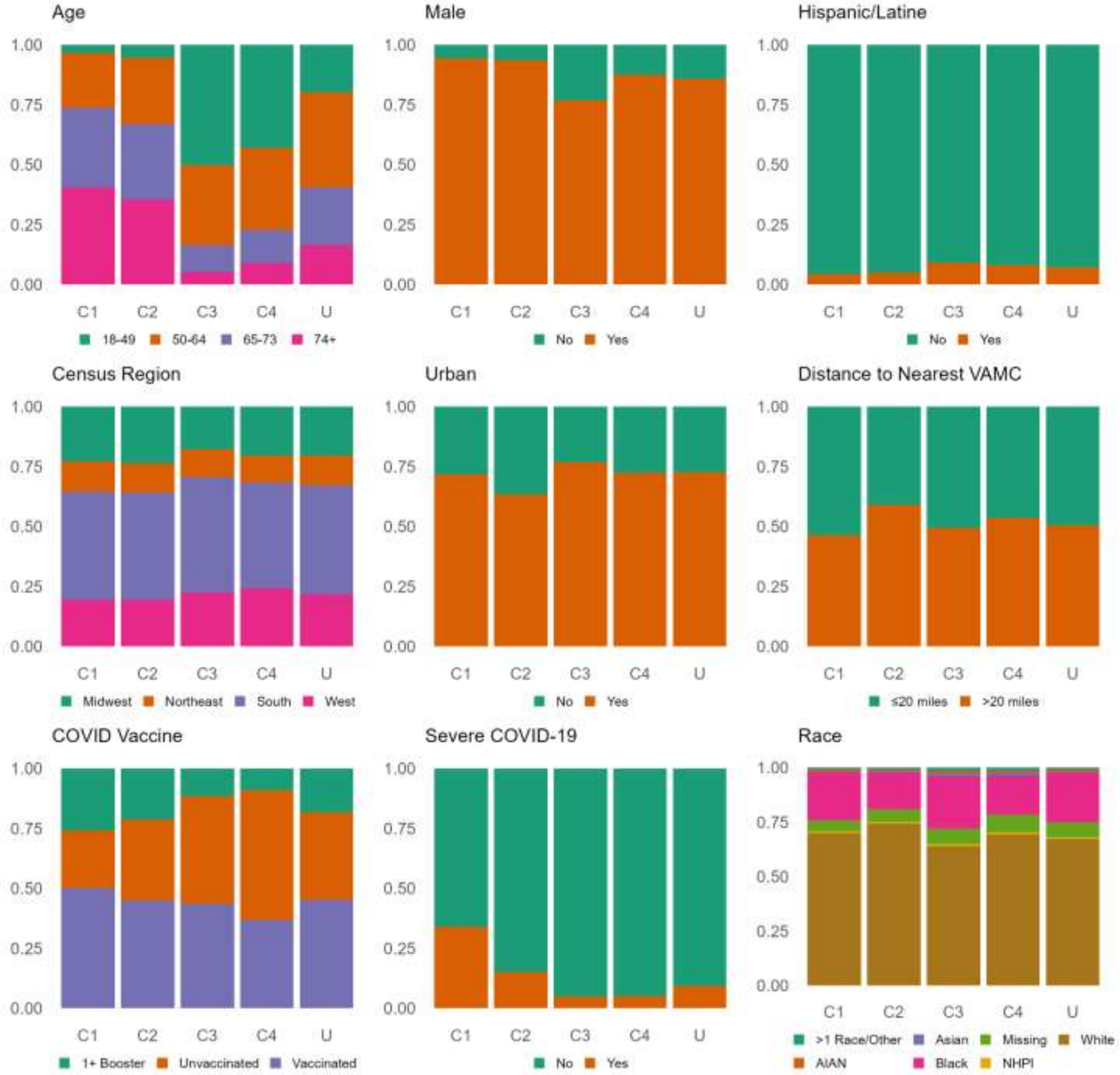

Note: C1 = Class 1 (n=46,693), C2 = Class 2 (n=66,359), C3 = Class 3 (n=82,309), C4 = Class 4 (n=86,208), U = Unclassified (n=3,666)

© 2025 Bui DP et al. *JAMA Network Open*.

eFigure 5. Comorbidity indices and healthcare utilization patterns of patients by four latent class solution.

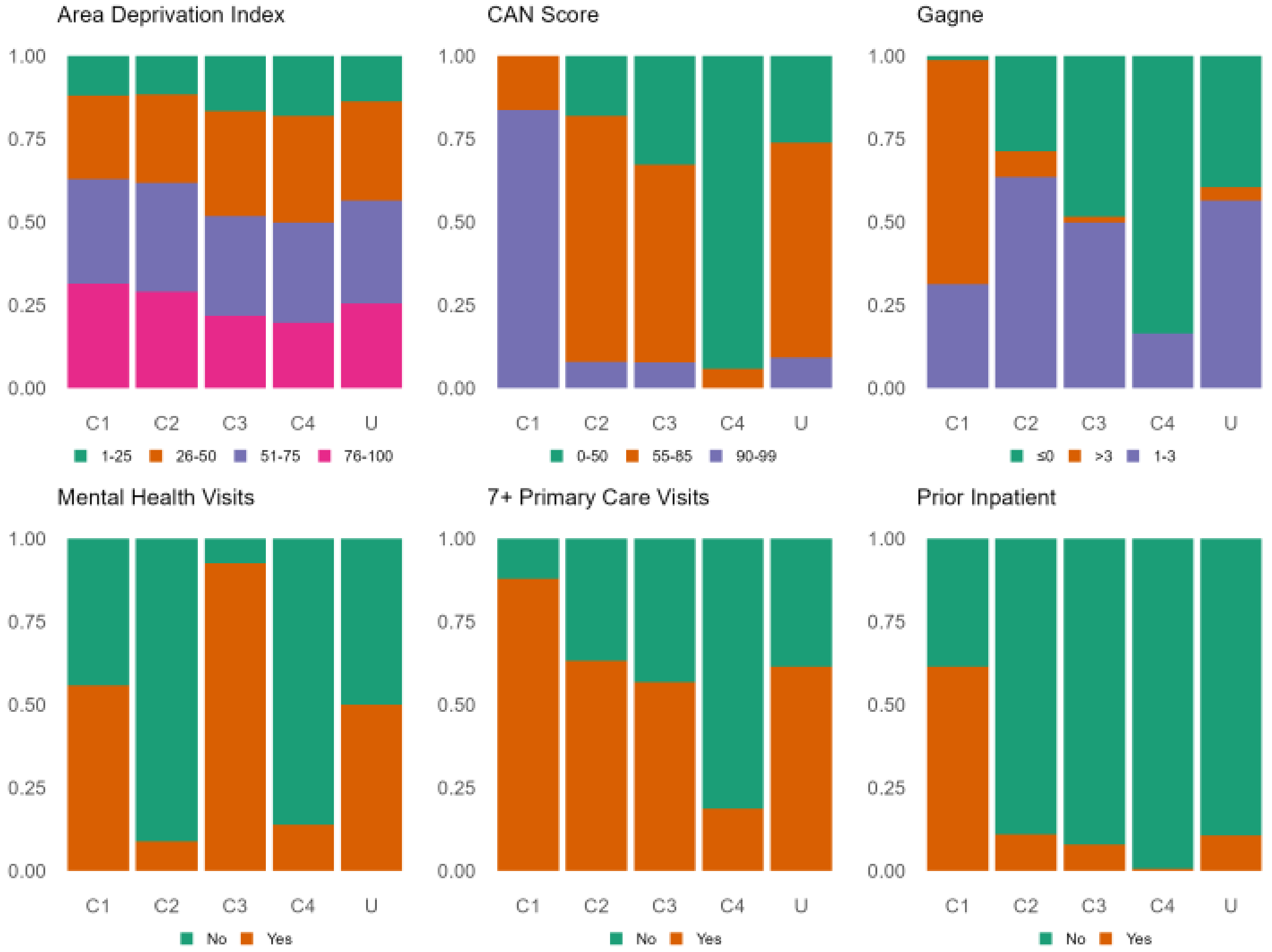

Note: C1 = Class 1 (n=46,693), C2 = Class 2 (n=66,359), C3 = Class 3 (n=82,309), C4 = Class 4 (n=86,208), U = Unclassified (n=3,666)

© 2025 Bui DP et al. *JAMA Network Open*.

eFigure 6. Comorbidities of patients stratified by four latent class solution.

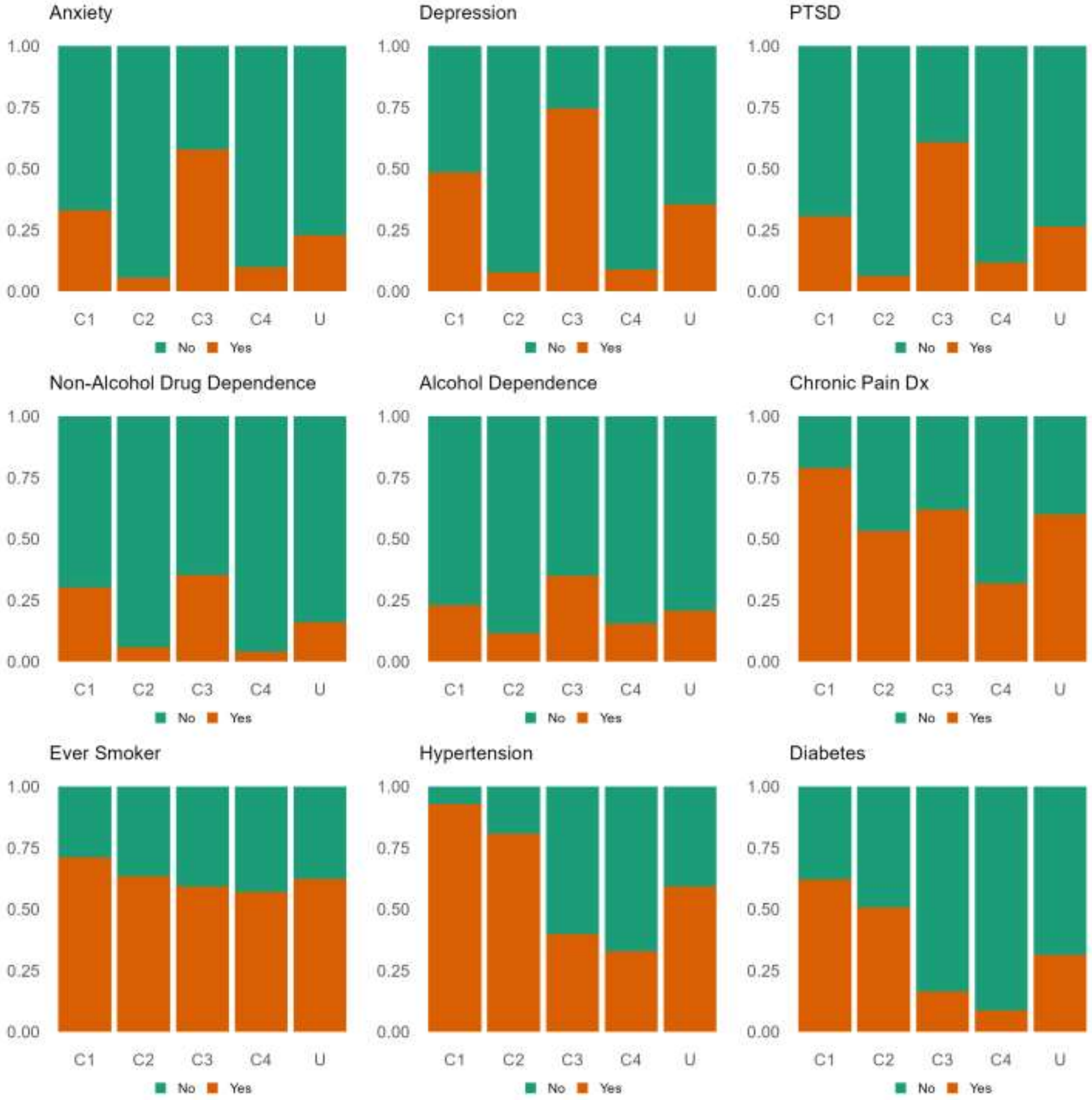

Note: C1 = Class 1 (n=46,693), C2 = Class 2 (n=66,359), C3 = Class 3 (n=82,309), C4 = Class 4 (n=86,208), U = Unclassified (n=3,666)

© 2025 Bui DP et al. *JAMA Network Open*.

eFigure 7. Heatmap of crude risk of suicide attempt and other self-directed violence (SDV) per 10,000 Veterans after COVID-19 infection, stratified by sociodemographic variables and other characteristics.

	1-Month Outcomes					6-Month Outcomes					12-Month Outcomes				
**Characteristic**	**C1**	**C2**	**C3**	**C4**	**U**	**C1**	**C2**	**C3**	**C4**	**U**	**C1**	**C2**	**C3**	**C4**	**U**
**Age Group (years)**															
18-49	96.8	5.6	32.5	6.5	13.8	283.9	11.1	128.3	21.3	27.5	451.6	25.0	207.2	38.6	27.5
50-64	39.4	2.7	23.2	2.4	0.0	133.1	9.8	87.3	7.8	20.7	207.1	17.5	141.3	14.2	48.2
65-73	16.8	2.4	10.9	0.8	0.0	51.0	6.7	52.3	3.3	0.0	87.2	10.0	86.1	3.3	11.3
74+	6.8	1.7	11.6	3.9	0.0	19.5	3.4	13.9	6.6	16.6	30.5	6.0	41.7	6.6	16.6
**Sex**															
Female	23.2	2.4	17.3	2.8	0.0	96.6	7.1	97.2	11.1	0.0	154.6	14.3	159.2	28.8	19.3
Male	20.4	2.4	28.4	4.2	3.2	62.8	6.6	101.0	13.1	19.1	100.7	11.3	164.1	21.6	31.8
**Race**															
AI/AN	0.0	0.0	11.3	0.0	0.0	110.2	17.9	158.2	0.0	0.0	192.8	17.9	180.8	0.0	0.0
Asian	0.0	0.0	58.2	0.0	0.0	51.3	20.5	133.1	26.2	0.0	102.6	20.5	191.3	32.8	0.0
Black	29.2	1.8	25.3	3.3	12.2	93.5	9.0	85.0	11.1	12.2	140.3	14.4	143.7	26.2	36.5
NH/PI	103.9	0.0	11.1	0.0	0.0	103.9	16.9	44.4	11.1	0.0	103.9	16.9	111.0	44.4	0.0
White	16.5	2.8	25.8	4.7	0.0	53.2	6.1	101.3	13.0	16.2	91.1	10.6	165.7	20.6	28.4
Multiracial	48.4	0.0	50.9	0.0	0.0	169.5	0.0	135.7	19.9	0.0	169.5	36.1	186.6	29.9	0.0
Unknown	25.2	0.0	20.9	2.9	0.0	67.3	2.6	127.1	12.9	39.8	92.5	7.9	198.5	27.2	39.8
**Hispanic/Latine**															
No	20.6	2.5	27.0	3.9	2.9	64.7	6.8	103.4	13.0	14.7	104.5	11.4	166.7	23.0	26.5
Yes	19.8	0.0	14.9	5.7	0.0	64.3	3.1	66.3	11.5	37.6	84.1	12.4	124.5	17.2	75.2
**Census Region**															
Northeast	15.2	2.5	23.0	3.0	0.0	57.4	3.7	97.1	13.2	43.9	96.2	7.4	149.4	15.2	43.9
Midwest	18.9	1.9	28.0	5.1	13.4	55.7	7.1	117.0	14.3	13.4	95.3	12.2	199.8	22.3	40.3
South	23.2	2.7	23.7	3.4	0.0	67.2	8.1	88.0	12.4	0.0	106.9	11.8	141.2	22.9	12.0
West	19.9	2.3	30.3	4.8	0.0	74.1	4.6	114.3	12.5	37.7	110.7	12.4	187.5	25.4	50.3
**Urban Residence**															
No	10.6	2.9	25.1	3.4	0.0	37.8	6.2	88.4	10.5	0.0	58.3	12.0	148.1	17.7	0.0
Yes	24.5	2.1	26.1	4.3	3.8	75.3	6.9	103.6	13.8	22.5	121.6	11.2	167.4	24.3	41.3
**Distance to nearest Veteran Health Administration Medical Center (miles)**															
≤20 miles	24.4	3.3	29.8	4.8	0.0	82.2	8.1	107.3	13.5	11.1	132.5	14.0	169.2	24.0	22.1
>20 miles	16.2	1.8	21.8	3.5	5.4	44.4	5.6	92.8	12.3	21.5	70.2	9.7	156.6	21.2	37.7
**COVID-19 Vaccination**															
Unvaccinated	16.1	3.1	28.1	4.9	7.5	60.9	8.0	103.8	14.8	22.5	104.8	14.2	170.3	26.5	37.5
Vaccinated	25.2	3.0	27.4	3.5	0.0	71.6	7.4	105.2	11.6	12.0	111.3	12.1	169.2	18.9	30.1

© 2025 Bui DP et al. *JAMA Network Open*.

	1-Month Outcomes					6-Month Outcomes					12-Month Outcomes				
**Characteristic**	**C1**	**C2**	**C3**	**C4**	**U**	**C1**	**C2**	**C3**	**C4**	**U**	**C1**	**C2**	**C3**	**C4**	**U**
1+ Booster	15.7	0.0	11.6	1.3	0.0	54.6	2.8	66.4	6.5	14.9	87.8	5.7	110.7	13.0	14.9
**Severe COVID-19**															
No	19.4	2.0	20.7	3.9	3.0	65.5	6.6	90.2	12.7	18.0	104.0	11.5	151.6	22.1	33.0
Yes	22.7	5.0	127.0	6.9	0.0	63.2	7.0	293.9	16.2	0.0	102.9	11.0	383.6	30.0	0.0
**Area Deprivation Index**															
1-25	24.2	2.6	25.4	3.3	0.0	50.3	6.6	102.9	10.5	60.5	81.9	13.2	158.9	26.1	60.5
26-50	16.7	1.7	22.8	2.9	0.0	79.8	4.0	94.4	12.9	9.3	118.4	9.3	159.4	20.9	18.7
51-75	16.9	1.9	25.3	5.1	0.0	50.0	6.6	103.1	12.1	9.0	92.3	11.3	168.9	21.1	26.9
76-100	24.6	3.2	31.2	4.8	10.9	68.0	8.4	102.0	15.0	10.9	104.5	12.7	159.2	22.8	32.7
Unknown	33.8	7.4	29.7	6.9	0.0	108.1	14.9	101.0	27.8	0.0	168.9	14.9	201.9	34.7	0.0
**1-year Hospitalization/Mortality Care Assessment Needs (CAN) Score**															
0-50	0.0	1.7	19.8	4.0	10.5	0.0	3.3	60.0	12.8	10.5	0.0	8.4	102.5	21.9	31.6
55-85	5.3	2.7	20.5	3.9	0.0	22.6	7.4	88.2	13.8	21.2	38.5	12.3	144.7	31.6	34.0
90-99	23.2	1.9	87.1	NA	0.0	72.2	7.5	343.7	NA	0.0	115.5	11.3	541.2	NA	0.0
Unknown	61.4	0.0	127.0	11.3	0.0	153.4	0.0	381.0	11.3	0.0	214.7	0.0	412.7	22.6	0.0
**Gagne Score**															
≤0 (better health)	47.9	3.7	17.6	4.2	6.9	111.8	6.8	74.1	13.1	6.9	159.7	12.6	122.9	22.6	27.6
1-3	20.5	1.9	32.8	3.5	0.0	70.5	7.1	119.0	12.0	19.3	110.2	11.8	192.2	21.8	29.0
4+ (poorer health)	20.0	2.0	56.5	NA	0.0	61.0	2.0	282.3	NA	69.0	99.5	3.9	437.5	NA	69.0
**≥1 Mental Health Visit (12 months)**															
No	2.9	2.0	4.9	3.4	5.5	7.3	5.3	21.4	9.3	10.9	13.6	9.6	41.2	16.2	21.8
Yes	34.5	6.7	27.5	8.3	0.0	110.0	20.2	106.4	34.9	21.8	174.8	30.3	172.6	61.4	38.2
**≥7 Primary Care Visits (24 months)**															
At least 7	19.5	1.9	18.2	2.5	4.4	61.0	6.0	87.4	8.7	13.3	96.3	9.1	145.4	16.1	22.2
Less than 7	28.1	3.3	36.0	4.4	0.0	91.4	7.8	116.8	13.9	21.2	156.4	15.5	186.0	24.0	42.4
**≥1 Prior Hospitalization (12 months)**															
No	14.5	2.4	24.2	4.1	0.0	42.3	6.3	93.2	12.9	15.3	72.9	10.7	149.8	22.4	30.5
Yes	24.4	2.7	45.7	0.0	25.6	78.7	9.6	179.8	16.5	25.6	122.9	17.9	313.9	33.1	25.6
**Anxiety Diagnosis**															
No	9.6	2.1	23.9	4.2	3.5	30.7	6.2	76.9	12.1	21.2	49.9	11.0	128.7	21.3	38.9
Yes	42.8	8.2	27.3	2.4	0.0	133.7	13.7	117.1	20.2	0.0	212.9	19.1	187.9	33.3	0.0
**Depression Diagnosis**															
No	5.8	2.1	14.3	4.1	4.2	16.7	6.0	55.4	11.3	12.7	25.8	10.3	98.4	19.7	25.4
Yes	36.2	5.8	29.8	4.0	0.0	115.6	13.5	115.4	29.1	23.0	186.1	25.1	185.0	51.5	38.3
**PTSD Diagnosis**															
No	7.7	2.6	21.7	3.5	3.7	32.1	6.6	75.4	11.2	22.2	54.3	11.4	121.0	19.2	40.8

© 2025 Bui DP et al. *JAMA Network Open*.

	1-Month Outcomes					6-Month Outcomes					12-Month Outcomes				
Characteristic	C1	C2	C3	C4	U	C1	C2	C3	C4	U	C1	C2	C3	C4	U
Yes	49.8	0.0	28.6	8.0	0.0	138.8	7.3	116.0	25.9	0.0	216.0	12.2	189.9	47.8	0.0
**Bipolar Diagnosis**															
No	13.8	2.1	21.6	3.9	2.9	45.7	5.8	84.5	11.7	17.3	71.8	10.4	137.2	20.8	28.8
Yes	115.8	25.8	69.1	20.3	0.0	331.3	77.3	258.9	111.8	0.0	550.0	103.1	424.3	172.8	52.4
**Schizophrenia Diagnosis**															
No	15.3	2.4	23.8	4.1	2.8	50.6	6.4	93.2	12.7	14.2	83.5	11.0	152.7	22.3	25.5
Yes	124.6	0.0	93.6	0.0	0.0	342.5	31.9	325.6	72.7	70.4	502.7	63.9	496.5	72.7	140.8
**Non-alcohol substance use disorder**															
No	6.7	2.4	13.9	3.7	0.0	19.0	6.2	59.3	12.3	13.0	33.4	10.6	96.5	21.7	29.2
Yes	52.5	2.6	47.9	11.8	17.1	170.3	13.0	174.9	26.5	34.1	266.1	25.9	284.8	41.2	34.1
**Alcohol Dependence**															
No	8.9	2.4	16.1	3.8	0.0	28.9	6.5	71.0	11.8	13.8	47.0	11.1	111.9	21.4	27.6
Yes	59.6	2.6	44.1	5.2	13.1	184.3	7.8	154.2	18.6	26.2	293.2	14.3	257.7	28.3	39.3
**Ever Smoker**															
No	15.6	2.9	18.8	3.8	0.0	50.4	6.6	77.8	10.8	14.5	76.3	11.1	122.3	20.2	36.2
Yes	22.6	2.1	30.7	4.3	4.4	70.5	6.6	115.4	14.5	17.5	114.8	11.6	190.8	24.2	26.2
**Chronic Pain**															
No	10.1	2.9	30.0	4.1	6.8	30.4	7.4	110.9	13.0	27.3	58.8	12.6	171.7	22.3	41.0
Yes	23.4	2.0	23.4	4.0	0.0	73.9	5.9	93.5	12.7	9.1	115.7	10.5	157.5	22.9	22.7
**Diabetes**															
No	28.3	2.8	28.3	4.2	4.0	85.5	6.4	104.6	13.2	11.9	129.7	10.7	171.5	23.0	31.8
Yes	15.8	2.1	13.4	2.7	0.0	52.0	6.8	77.3	9.4	26.1	87.8	12.2	118.9	17.5	26.1
**Hypertension**															
No	35.7	3.1	30.3	4.5	0.0	130.8	5.5	112.6	15.6	13.4	208.1	10.2	183.8	28.0	13.4
Yes	19.4	2.2	19.2	3.2	4.6	59.5	6.9	81.3	7.4	18.4	95.5	11.8	131.4	11.3	41.4
**Coronary Heart Disease**															
No	27.2	1.8	25.9	4.1	3.5	97.6	5.5	102.1	13.0	17.5	159.0	10.0	167.0	22.8	35.1
Yes	18.8	3.2	25.3	2.7	0.0	55.8	8.1	80.0	10.9	12.3	88.7	13.4	122.7	16.3	12.3
**Chronic Kidney Disease**															
No	22.9	3.0	25.2	4.0	3.3	83.4	7.1	99.7	13.0	16.6	137.4	13.4	162.8	22.8	26.6
Yes	19.4	1.0	31.7	5.5	0.0	55.2	5.5	103.5	11.1	15.2	86.5	7.0	164.4	16.6	45.6
**Pulmonary**															
No	20.7	2.5	25.5	4.2	3.6	58.6	6.1	100.9	13.3	21.4	97.1	11.1	164.9	22.8	39.2
Yes	20.5	2.3	27.8	2.0	0.0	69.5	8.0	96.1	6.0	0.0	108.9	12.6	152.4	18.0	0.0
**Cancer**															

© 2025 Bui DP et al. *JAMA Network Open*.

	1-Month Outcomes					6-Month Outcomes					12-Month Outcomes				
**Characteristic**	**C1**	**C2**	**C3**	**C4**	**U**	**C1**	**C2**	**C3**	**C4**	**U**	**C1**	**C2**	**C3**	**C4**	**U**
No	25.7	2.3	26.5	4.2	3.1	78.5	6.6	102.1	13.2	15.5	128.6	12.0	166.0	22.8	31.0
Yes	11.0	2.9	16.6	0.0	0.0	39.1	6.6	68.5	0.0	22.9	57.5	9.5	114.2	9.8	22.9
**Congestive Heart Failure**															
No	27.0	2.4	25.9	4.1	2.8	86.1	6.9	100.1	12.8	17.0	132.7	11.6	163.1	22.4	31.1
Yes	12.8	2.9	17.2	0.0	0.0	38.8	4.4	103.1	357.1	0.0	68.6	10.2	137.5	357.1	0.0
**Liver Disease**															
No	17.7	2.6	25.9	4.1	3.0	58.6	6.6	100.6	13.0	11.9	91.9	11.4	162.9	22.7	26.7
Yes	33.9	0.0	25.9	3.8	0.0	93.1	6.9	94.0	7.6	66.4	158.4	12.1	163.7	15.2	66.4
**Stroke/Cerebrovascular**															
No	21.3	2.3	26.1	4.1	2.9	65.1	5.9	100.7	12.9	17.2	102.3	10.6	163.4	22.5	31.5
Yes	17.6	3.9	15.4	0.0	0.0	62.8	15.6	76.9	13.9	0.0	109.1	21.5	143.6	27.8	0.0
**Dementia**															
No	21.3	2.5	26.3	4.1	2.8	66.6	6.9	100.2	12.9	17.1	104.9	11.9	163.2	22.6	31.3
Yes	16.3	0.0	0.0	0.0	0.0	53.2	0.0	94.1	0.0	0.0	96.0	0.0	144.8	0.0	0.0

Caption: Abbreviations: C1 = Class 1, C2 = Class 2, C3 = Class 3, C4= Class 4, U = Unclassified, AI/AN = American Indian/Alaska Native, NH/PI = Native Hawaiian/Pacific Islander.

© 2025 Bui DP et al. *JAMA Network Open*.

eFigure 8. Unadjusted rates of all outcomes, including suicide attempt, other self-directed violence (SDV), and death by latent classes and overall. Rates for Suicide attempt and SDV are per 10,000 Veterans.
Rates for death and no event are per 100 Veterans.

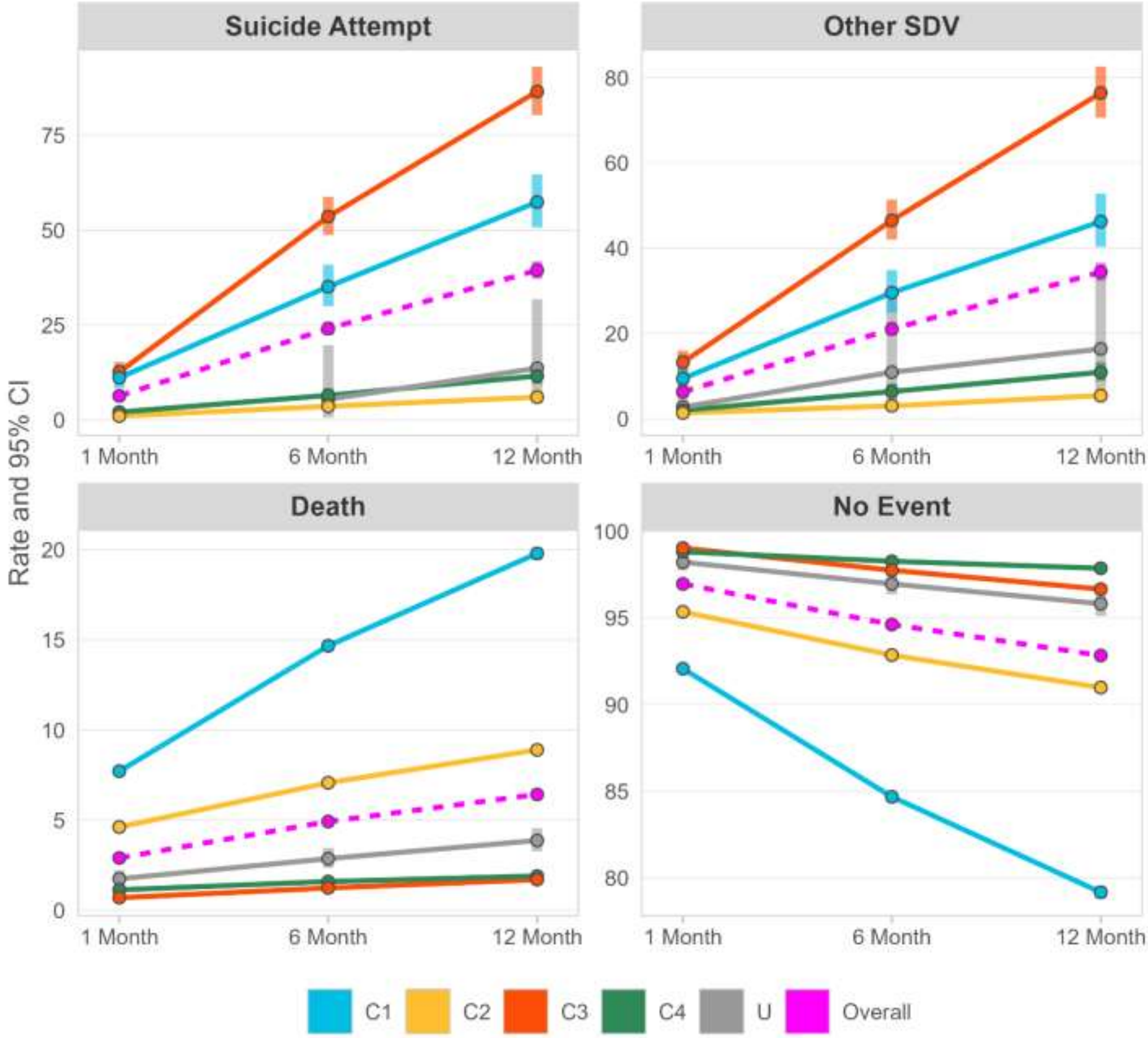

© 2025 Bui DP et al. *JAMA Network Open*.

eFigure 9. Sensitivty analysis: outcome rates by latent classes among those who survive outcome timeframes.
Rates for Suicide attempt and SDV are per 10,000 Veterans. Rate for no event are per 100 Veterans.

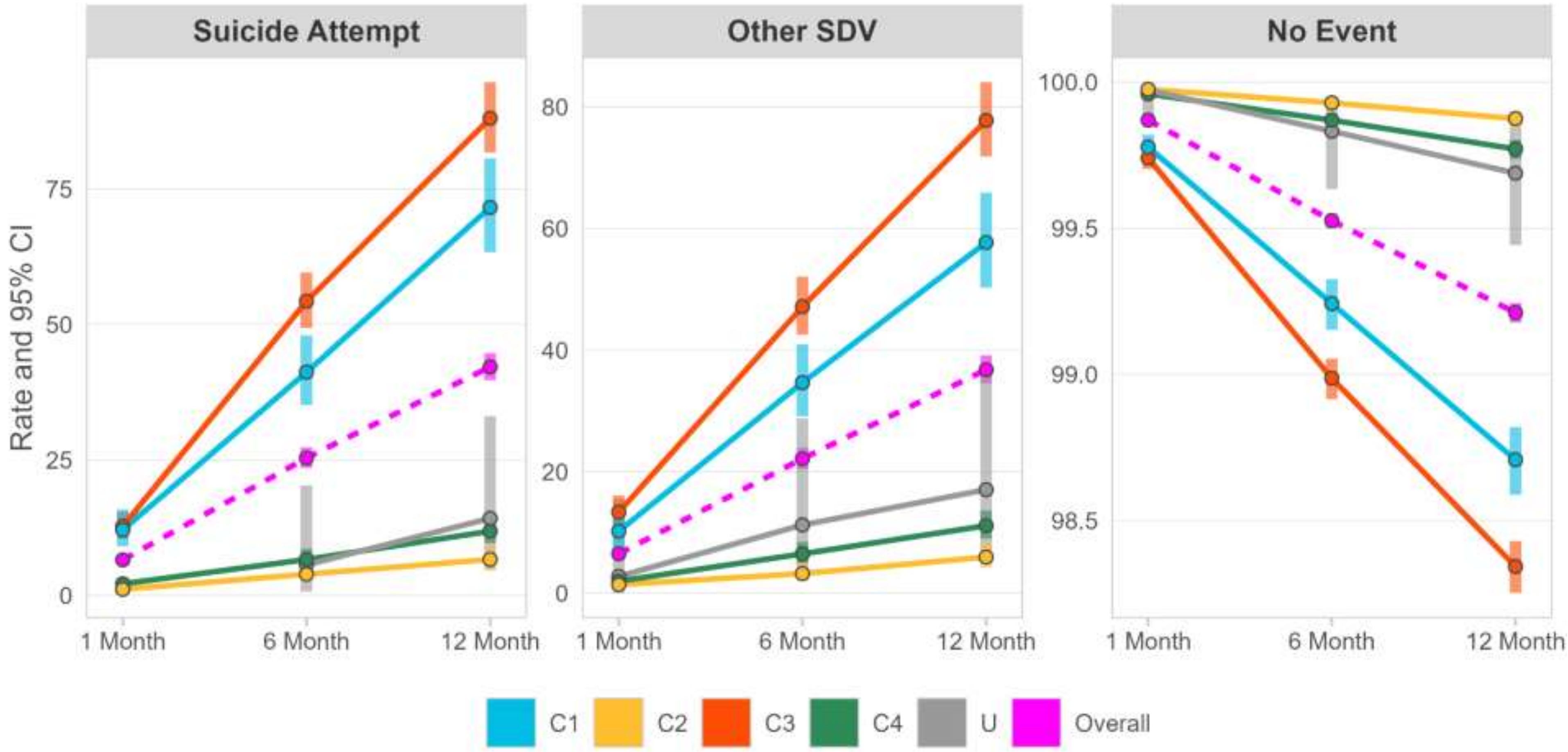

© 2025 Bui DP et al. *JAMA Network Open*.

eAppendix. Reporting on mortality outcomes across latent classes.

The 12-month mortality rate was 6.4% (CI: 6.3, 6.5) in the overall cohort (See **Figure 2** and **eTable4**). Class 1 Veterans had the highest 12-month mortality rate of 19.8% (CI: 19.4, 20.2), followed by Class 2 Veterans (8.9%, CI: 8.7, 9.1). Despite having the highest 12-month risk of suicide attempt and SDV, Class 3 Veterans had the lowest 12-month mortality rate (1.7%, CI: 1.6, 1.8) which was 91% lower than Class 1 (RR: 0.09, CI: 0.08, 0.21), 81% lower than Class 2 (RR: 0.19, CI: 0.18, 0.21) Veterans (**eTable 5**). Class 4 Veterans also had a low 12-month mortality rate (1.9%, CI: 1.8, 2.0) that was slightly higher than Class 3 Veterans (RR: 1.12, CI: 1.01, 1.24) but significantly lower than Class 1 (RR: 0.10, CI: 0.09, 0.10) and Class 2 (RR: 0.21, CI: 0.20, 0.23) Veterans.

© 2025 Bui DP et al. *JAMA Network Open*.
