# Supplementary material for: Veterans at High Risk for Post–COVID-19 Suicide Attempts or Other Self-Directed Violence
Source: JAMA Netw Open. 2025 Mar 4;8(3):e250061. doi: 10.1001/jamanetworkopen.2025.0061 (PMC11880954; doi:10.1001/jamanetworkopen.2025.0061)
Supplement: Supplement 2. — Data Sharing Statement [file jamanetwopen-e250061-s002.pdf]

Data Sharing Statement

Bui. Veterans at High Risk for Post–COVID-19 Suicide Attempts or Other Self-Directed Violence. *JAMA Netw Open*. Published March 04, 2025.
doi:10.1001/jamanetworkopen.2025.0061

Data
Data available: No
